# RNA modifications shape innate immunity and cellular adaptation during bacterial respiratory infection

**DOI:** 10.3389/fimmu.2026.1845134

**Published:** 2026-06-05

**Authors:** Martina M. Ivanova, Petya A. Dimitrova, Milena N. Leseva

**Affiliations:** Department of Immunology, Laboratory of Experimental Immunotherapy, Stephan Angeloff Institute of Microbiology, Sofia, Bulgaria

**Keywords:** epigenetic regulation, *in vitro* models, innate immune memory, innate immune response, lower airway infection, *Pseudomonas aeruginosa*, RNA modifications

## Abstract

Gram-negative bacteria are clinically significant pathogens responsible for life-threatening infections, including respiratory infections. These can be acute or persistent and can exacerbate existing chronic diseases, such as cystic fibrosis, COPD and lung cancer. In this review, we use *Pseudomonas aeruginosa* as a model organism that demonstrates the molecular complexity of host-pathogen interactions during lower airway infection. Specifically, we focus on RNA modifications and show that they, on the one hand, regulate bacterial fitness and pathogenicity, and on the other control the execution of an effective host innate immune response. Furthermore, we examine the role of epigenetic and epitranscriptomic modifications in the immune dysregulation observed in sepsis, with an emphasis on sepsis-induced lung injury. Innate immune memory - a cellular adaptation mechanism to primary microbial stimulation - results in training or tolerization of host cells towards secondary immune challenges. While fundamentally grounded in epigenetic and metabolic reprogramming, we propose that it can crosstalk with epitranscriptomic regulation. To overcome limitations imposed by animal models when investigating microbe-induced epitranscriptomic dynamics, we highlight physiologically-relevant *in vitro* tissue models that can complement work performed *in vivo*. Ultimately, a detailed understanding of the RNA modification landscape regulating host-pathogen interactions will help us identify new therapeutic targets and molecular pathways to better manage the clinical symptoms of bacterial respiratory infections and address the growing challenge of antimicrobial resistance.

## Introduction

Bacterial infections pose a major challenge to healthcare systems around the world. Making matters worse, evolutionary adaptation and the emergence of antimicrobial resistance (AMR) has rendered many bacteria insensitive to traditional antibiotics. Among Gram-negative bacteria, resistance to carbapenems has increased most significantly in the past few decades (1). In 2021, an estimated 4.71 million deaths globally [95% uncertainty interval (UI), 4.23-5.19] were associated with persistent antibiotic resistance, including 1.14 million (approx. 24%) directly attributable to bacterial AMR (1). That same year, 21.35 million people globally died from sepsis (1), a complication of infectious diseases associated with acute systemic inflammation, multi-organ failure, high morbidity and mortality. It is estimated that by 2050, 11.1 million deaths [ranging between 9.08-13.20] from bacterial AMR can be avoided by the development of Gram-negative drug pipelines for new classes of antimicrobial agents (1). These astonishing numbers emphasize the importance of studies on bacterial virulence and the causes of AMR, as well as of the development of new approaches to overcome them. A successful campaign against bacterial infections ultimately comes down to the in-depth understanding of the various evolving mechanisms these pathogens use to: invade their hosts; outcompete prokaryotic rivals; mimic host self-structures and hide from the immune system through structural transformations, such as biofilm formation, cell internalization and immunosuppression. In this Review, we demonstrate that these strategies intersect with dynamic host cell signaling and innate immune defenses and are regulated at the epigenetic and epitranscriptomic levels. Such molecular mechanisms, therefore, constitute viable targets for the development of alternative antimicrobial therapeutics.

Covalent modifications occur in all classes of RNA molecules (ribosomal, transfer, messenger, and non-coding RNA) and are collectively referred to as the cell’s epitranscriptome. Over 170 RNA modifications have been described to date and can be found in the MODOMICS database (2). RNA modifications can have structural and functional roles. For example, tRNAs are extensively modified during their post-transcriptional processing, and modifications placed in the core region are important for stability and 3D folding, while those in the anticodon loop region play a role during translation. Likewise, internal mRNA modifications have well established roles in the regulation of transcript half-life and decay, but also in transcript export out of the nucleus and in the regulation of translation efficiency. More recently, certain modifications (e.g. N6-methyladenosine) have also been shown to act as regulators of the chromatin state, genomic stability and transcription. Notably, RNA modifications are dynamic and their levels, types and positioning change during cellular differentiation, the transition from cell homeostasis to functional activation, cellular adaptation to stress and nutrient availability, as well as during infection. This is possible due to the action of dedicated enzymes that place the respective modifications and others which remove them. On the one hand, modifications of rRNA, tRNA and mRNA are used by pathogens to successfully establish and maintain an infection, including through resistance to xenobiotics, and on the other by the host cells in order to eliminate the pathogen and mount an effective immune response. Dysregulation of epitranscriptomic and epigenetic modifications (e.g. histone post-translational modifications; PTMs), as well as their crosstalk, can result in exacerbated infectious disease due to hyper-inflammatory, exhausted or muted immune responses to invading pathogens, which frequently lead to tissue damage and long-term morbidity.

According to the WHO Bacterial Priority Pathogens List (BPPL) for 2024, high-priority respiratory pathogens include carbapenem-resistant *Pseudomonas aeruginosa* (3). In this narrative Review, we focus on bacterial infection of the lower airways and in particular use *Pseudomonas aeruginosa* as a case study that illustrates complex host-pathogen interactions at the molecular level. This microbe was chosen because of: i) its relatively well-characterized epitranscriptome; ii) recently reported high-throughput *P. aeruginosa* tRNA and mRNA epitranscriptome mapping advances; and iii) its higher priority status on the BPPL compared to other respiratory bacteria frequently used in RNA modification studies. We searched PubMed for articles published from 2021 to 2026 using the terms [Pseudomonas aeruginosa infection] AND [RNA modification]. Studies which focused on “modification” in the context of optimized antimicrobial agents or post-translational modification of bacterial proteins were excluded. We included original research that demonstrated epitranscriptomic regulation of bacterial physiology during infection (i.e. pathogenicity, stress response, metabolism and antimicrobial resistance), as well as host innate immune response to bacterial infection. We also highlight physiologically-relevant non-animal models of *P. aeruginosa* respiratory infection reported in studies published in the last 5 years.

## *Pseudomonas aeruginosa* and lower respiratory tract infection

Bacterial infections of the lower respiratory tract can be mild (bronchitis, bronchiolitis) or severe (pneumonia, sepsis). Severe infections are accompanied by acute inflammation with the development of acute lung injury/inflammation (ALI) or acute respiratory distress syndrome (ARDS) in critically ill patients and continue to be a leading cause of morbidity and mortality. Among the many clinically important respiratory pathogens, *Pseudomonas aeruginosa* stands out as a highly adaptable, ubiquitous and opportunistic Gram-negative bacterium. It is a common cause of nosocomial infections, particularly community-acquired and ventilator-associated pneumonia and pleural-space infections, affecting the immunocompromised and those with chronic lung diseases such as cystic fibrosis (CF) and chronic obstructive pulmonary disease (COPD). In patients with CF, *P. aeruginosa* is a critically important pathogen (4) leading to recurring respiratory infections and acute exacerbations. It is also one of the most frequently isolated microbes from patients with non-CF bronchiectasis and is associated with a decline in lung function and increased mortality (5). A recent systematic review of studies that examined non-viral pulmonary infection in the context of lung cancer showed that among lung cancer patients *Pseudomonas aeruginosa* is one of the most common causes of pulmonary infections with a cumulative prevalence of 17.0%, second only to *Klebsiella pneumoniae* (6). Moreover, bacterial respiratory infections appear to play a significant modulating role in lung cancer pathogenesis through effects on inflammatory pathways such as Toll-like receptor 4 (TLR4)/MyD88 and Nuclear factor kappa B (NF-κB), and Programmed cell death protein-1 (PD-1)/PD-L1 signaling (6). Indeed, *Pseudomonas aeruginosa* lipopolysaccharide (LPS)-mediated chronic inflammation activates PD-1/PD-L1, induces T-cell exhaustion and creates an immunosuppressive microenvironment that stimulates lung tumorigenesis (7). Hence, the persistent inflammation and tissue injury associated with prolonged respiratory infections, as well as infection-induced airway remodeling combined with immunosuppression can promote lung cancer progression.

## Virulence factors and immune escape

Bacterial virulence factors and the state of the host immune system determine the severity, persistence and lethality of a *P. aeruginosa* infection. Virulence factors can be located on the cell surface, secreted in the environment (e.g. proteases and lipases), injected intracellularly by multi-protein secretion systems (T1, T2, T3 and T6 secretion systems), as well as can promote bacterial adaptation to the environment (quorum-sensing molecules). These bacteria employ a variety of immune escape mechanisms, which include: blocking of innate immune receptors and their downstream signaling; inhibition of the complement, opsonization and phagocytosis; induction of cell death through apoptosis and pyroptosis; degradation of cytokines; decreased antigen presentation and escape from the specific adaptive immune response (antibody recognition and protective Th1 immunity). Examples of surface and secreted virulence factors used by *P. aeruginosa*, as well as immune escape mechanisms have been non-exhaustively summarized in **Table 1**.

**Table 1:** Main virulence factors and defense/escape mechanisms of *Pseudomonas aeruginosa*.

Virulence factors		Recognition (PTI)/effect on host cells (effectors, ETI)	Immune escape mechanisms (10)
Surface virulence factors – Pattern recognition-triggered immunity (PTI)			
Exopolysaccharides (biofilm)	Psl Pel Alginate	TLR2/4 engagement on immune and epithelial cells; interaction with C-type lectins; initiation of ROS release	Protection from phagocytosis; resistance to oxidative stress; inhibition of complement deposition of C3b and opsonization
Porins	OprF	Bind C3b for opsonization; degraded by neutrophil elastase	Closed anchored conformation to escape immune recognition; bind IFN-γ; escape phagosomes
Lectins	LecB	Bind to glycosylated moieties of β1-intergrins and cause integrin endocytosis in epithelial cells	Clear integrins from the cell surface and block cell migration
Flagella	Flagellin FliC	TLR5 engagement on immune and epithelial cells; acts as effector through T3SS and activates NLRC4-type inflammasome	Loss of expression through mutation and subsequent selection; induction of pyroptosis; degradation by alkaline protease A and elastase (TLR5 escape)
Fibers for adhesion/motility	Type IV Pili; PilA	Interaction with adhesin; attachment to cells via glycol-conjugates; induction of SP-A-mediated membrane permeability and opsonization	Initiation of biofilm formation and immune avoidance
Fimbrial pilli	Fimbriae	Adherence to cells; block ROS that indirectly interferes with Chaperone-Usher mechanisms	Complement evasion; downregulated for ROS-driven adaptation to planktonic states
Secreted virulence factors – Effector-triggered immunity (ETI)			
Type II secretion system (T2SS) for export of exotoxin A (ExoA), elastases (LasA/LasB), and phospholipase C		Not directly recognized by the innate immune system, instead act indirectly as effectors; ExoA inhibits protein synthesis by ADP-ribosylation of eukaryotic elongation factor 2	Surface modification changes recognition by innate immune receptors; complement inactivation; promotes evasion of bacterial clearance and intracellular persistence; promotes persistent infection
Type III secretion system (T3SS) for direct injection of exotoxins (effectors) and their translocation through the host membrane		Activation of NLRC4 inflammasomes by PilA, RhsT, FliC, Pscl; bind to the NLR-family apoptosis inhibitory proteins (NAIPs) for oligomerization of NLRC4	Direct cytotoxicity; altered cytoskeletal integrity; induction of autophagy to downregulate NLRC4
Type VI secretion system (T6SS) contractile tail-like structure; VgrG2b protease; Lipases PldA/PldB		Not directly recognized by the innate immune system – act as effectors	Interference by VgrG2b with the host microtubule network and phospholipases; PldA/B hijack the PI3K/Akt pathway to facilitate bacterial entry; antagonism against commensal bacteria to affect tolerant mucosal immunity
Efflux pump for transport of phenazine - PumA		Not directly recognized by the innate immune system – act as effectors	Blocks NF-κB translocation and TLR signaling; interacts with UBAP1 and interferes with the ESCRT complex, and degradation of TNFR1 and integrins

## Heterogeneity and antibiotic resistance

The transition from acute to chronic infection requires bacterial adaptation that often results from mutation (for example, *mucA* mutations for overproduction of alginate or the transition from motile to immobile Type 3 secretion system-negative survival phenotype). Acquired and intrinsic antimicrobial resistance pose significant difficulties in clinical therapy. Multiple mechanisms contribute to the development of antibiotic resistance and these have been reviewed by Hu and Chua (8). They can include: i) upregulation of efflux pumps; ii) acquisition of resistance genes through horizontal gene transfer; and iii) biofilm formation. New alternative therapies are being developed to treat *P. aeruginosa* infections, including antimicrobial peptides, nanoparticles and quorum-sensing inhibitors, but they are yet to make their way into the clinic (8). Spatial transcriptomics of free-living bacteria and biofilm aggregations of *Pseudomonas aeruginosa* have recently revealed the extensive heterogeneity that exists within bacterial populations (9). Dar et al. developed parallel sequential fluorescence *in situ* hybridization (par-seqFISH) and used it to uncover, at the single-cell level, the various metabolic and virulence-related transcriptional states that were simultaneously present within the cultures (9). This functional heterogeneity and the existence of bacterial subpopulations should be kept in mind when investigating *P. aeruginosa* biology (discussed later in the section on non-animal models of respiratory infection).

## Immune response in *P. aeruginosa* respiratory infection

### Innate immune responses and host-pathogen interactions

**Figure 1:**
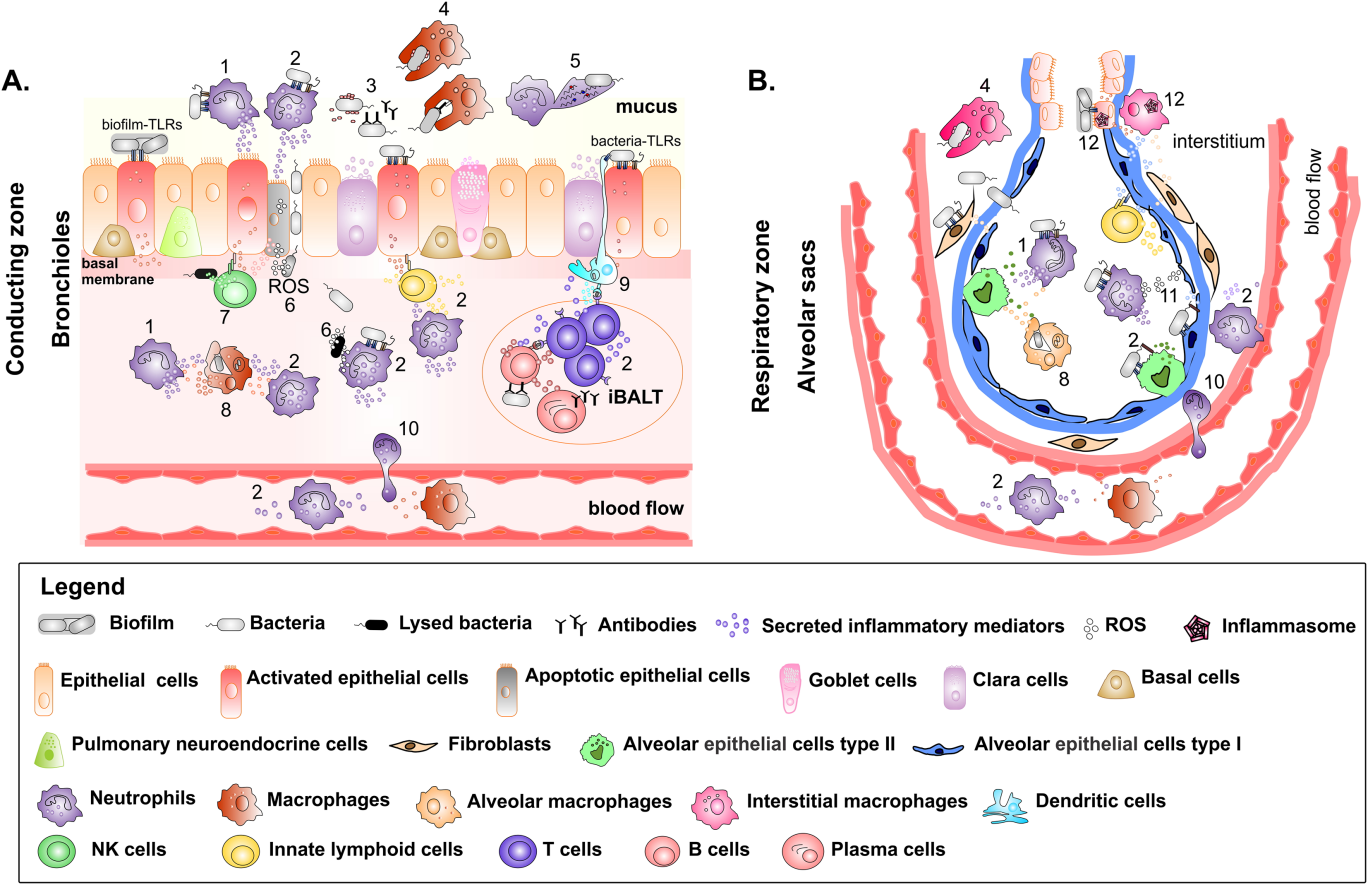
Immune response against *P. aeruginosa* lung infection in conducting **(A)** and respiratory zones **(B)**. The bacteria and bacterial biofilm are recognized by toll-like receptors (TLRs) on epithelial cells and trigger multiple effector functions in non-immune and immune cells: 1, degranulation; 2, inflammatory mediator/cytokine secretion; 3, C3b/Ab deposition; 4, opsonization; 5, netosis; 6, bacterial killing by NK cells; 7, ROS-mediated bacterial killing; 8, phagocytosis; 9, antigen presentation; 10, neutrophil recruitment; 11, ROS-mediated apoptosis; 12, pyroptosis.

Bacterial infections directly affect the structure of the lower respiratory tract at conducting zones (trachea, bronchi, bronchioles, and terminal bronchioles) and in the respiratory zones (respiratory bronchioles and alveolar sacs). The pathogens can damage the bronchial epithelium (**Figure 1A**), the basal membrane, the parenchyma, as well as the alveoli (**Figure 1B**) (11). The bacteria can also disturb various layers of the local immune system, including: innate defense mechanisms in the bronchial epithelium; interstitial macrophages and dendritic cells in the lung parenchyma; innate lymphoid cells (ILCs) located in alveolar capillary beds; inducible bronchus-associated lymphoid tissue (iBALT) with organized follicles of lymphocytes (T- and B-cell zones and dendritic cells) located near the basal membrane (see **Figure 1A**); local lymph nodes (T- and B-cells, and dendritic cells) and broncho-alveolar cells on the luminal side [mostly alveolar macrophages and rarely lymphocytes, as well as neutrophils, dendritic cells, and ILCs (during inflammation)] (**Figure 1**) (11). Spatial compartmentalization with pathogen-sensitive non-immune and immune cells allows the lung to function as an independent organ during infection. The immune response begins with the recognition of the bacteria by innate immune receptors (TLRs and NOD-like receptors; NLRs) on bronchial and alveolar epithelial cells, as well as on alveolar and interstitial macrophages and dendritic cells. Secretion of chemo-attractants and chemokines promotes neutrophil recruitment, and the release of pro-inflammatory cytokines such as tumor necrosis factor (TNF)-α, and interleukins IL-1β, IL-6, and IL-8. This creates an inflammatory environment to enhance bacterial killing, complement activation, netosis, opsonization and phagocytosis, and to recruit other immune cell types such as NK cells (releasing IFN-γ) and ILCs (releasing IFN-γ and IL-17) (**Figure 1**). The immune response can involve T lymphocytes, which differentiate to Th1 or Th17 effector cells and B-cells, which differentiate to plasma cells and produce secretory immunoglobulins IgA, IgM and IgG. At later stages of infection, anti-inflammatory cytokines are also produced (IL-22, IL-4, and IL-10), as well as soluble pro-inflammatory cytokine receptors (11). We refer the reader to a recent Editorial by Hussain et al. for a more detailed discussion of emerging concepts for mechanisms regulating the host-microbe crosstalk and immune responses (12).

### Pattern-recognition mechanisms of immunity against *P. aeruginosa* infection

**Figure 2:**
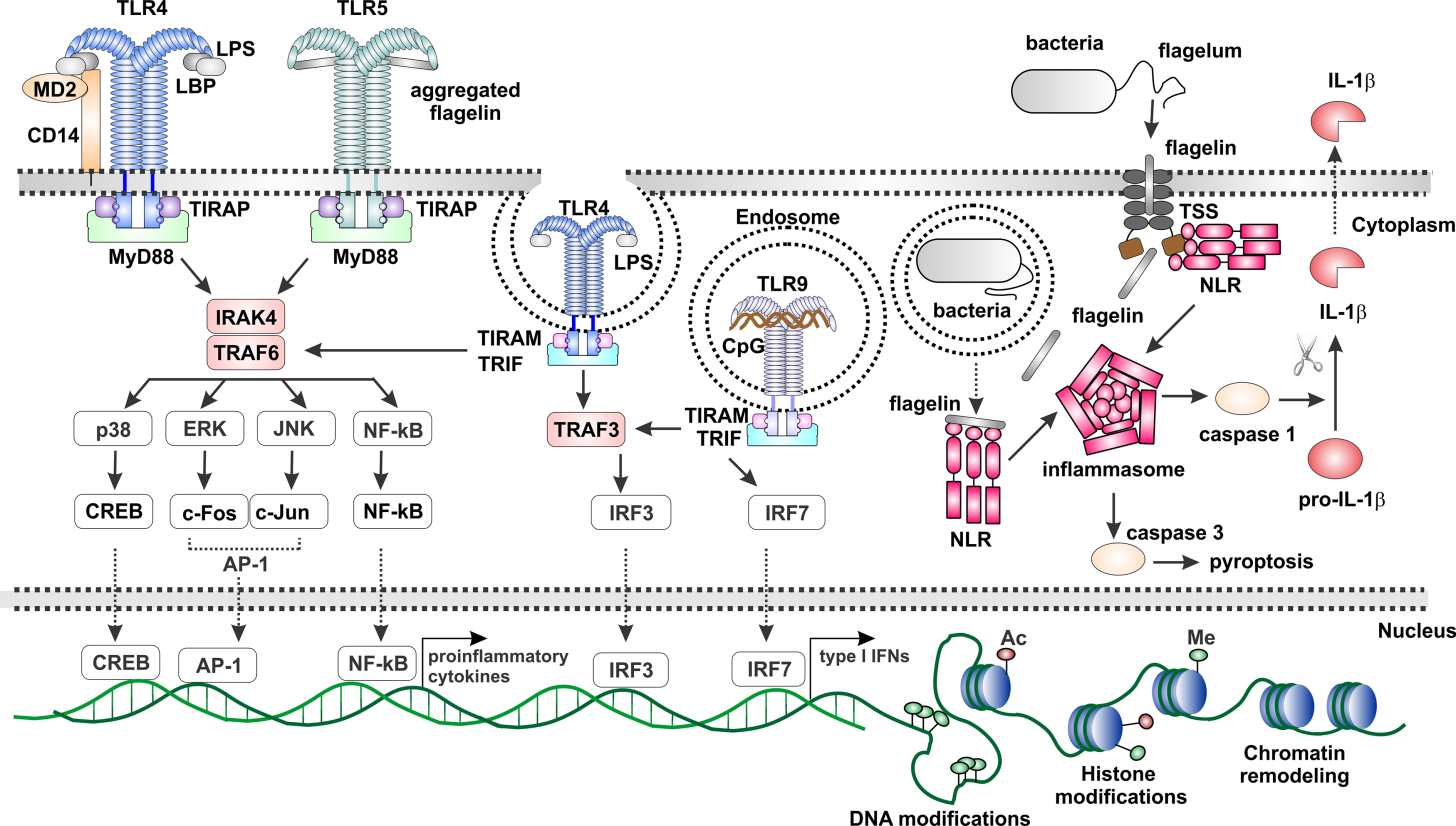
Pattern-recognition mechanisms of immunity during *P. aeruginosa* infection. TLRs and C-type lectin receptors on the cell surface (TLR4 for LPS/TLR5 for flagellin) or endosomal membranes (TLR9 for bacterial CpG), and NLRs sensing the bacterial TSS system or bacterial molecules in the cytoplasm. The engagement of a TLR recruits adaptor proteins (such as TIRAP or TIRAM, MyD88, IRAK4, and TRIF 3 and 6) and leads to the activation of multiple signaling pathways [p38 Mitogen-activated protein kinase (MAPK)/Extracellular signal-regulated kinases (ERK), AP-1 transcription factor complex, NF-kB and Interferon regulatory factor (IRF) 3 and 7], which induce pro-inflammatory mediators and/or type I interferons (IFNs). This process is regulated by chromatin remodeling, histone modification through methylation (Me) and acetylation (Ac), and DNA methylation. The activation of the inflammasome pathway results in activation of caspases (1 and 3) and cleavage of pro-IL-1β to active IL-1β.

Various membrane and cytosolic pattern recognition receptors (PRRs) are used by the host to sense bacterial molecules, collectively referred to as pathogen-associated molecular patterns (PAMPs). The TLRs and C-type lectin receptors are bound to surface or endosomal membranes, while the Nucleotide-oligomerization domain (NOD)-like receptors (NLRs) and Retinoic acid-inducible gene-like receptors (RIG-I) detect bacterial molecules in the cytoplasm. The engagement of a particular TLR can recruit specific adaptor proteins (such as TIRAP or TIRAM, MyD88, IRAK4 and TRIF3/6) and leads to the activation of multiple signaling pathways for the expression of pro-inflammatory mediators (**Figure 2**). The recognition of Gram-negative bacteria by NLRs can activate several host effector functions. For example, the cytoplasmic NLR receptor NOD1 recognizes γ-d-glutamyl-meso-diaminopimelic acid of Gram-negative bacteria, while NOD2 binds the muramyl dipeptide motif of the peptidoglycans of both Gram-negative and Gram-positive bacteria (13). NLRs are multimeric proteins with domains allowing their cytosolic oligomerization and formation of the inflammasome. The inflammasome activates the protease caspase-1 to cleave the inactive form of pro-IL-1β (**Figure 2**) and pro-IL-18 as well as the pore-forming protein gasdermin D (GSDMD) to active molecules leading to cytokine release, amplification of the inflammatory cascades and induction of cell death through pyroptosis (reviewed by McVey et al. (14)).

### Effector-triggered immunity

In addition to the traditional PAMP-sensing mechanisms of immunity, a new concept of effector-triggered immunity (ETI) has been introducd (15). ETI is defined as an immune reaction induced in response to the virulence-associated activity of pathogens, which produce proteins or effectors in the host cells that manipulate cellular processes in order to promote pathogen replication or transmission (see **Table 1** for examples). It can be triggered by targeting conserved host structures or mechanisms involved in the pathogenesis of the infection, such as the cytoskeleton, secretory pathways, autophagy, and inflammatory signaling pathways (see reviews by Remick et al. (15), as well as M. Orzalli and P. Parameswaran (16)). While PRR sensing is increased by both pathogens and commensal microbes, ETI distinguishes between harmful and harmless bacteria because it is induced only when pathogen effectors are produced. In addition, ETI can scale the magnitude and type of immune responses when commensal bacteria intensively colonize the host and transition into an invasive pathogen (see review by Vance et al. (17)). Pathogens can induce ETI-dependent stress and cell death contributing to the release of danger-associated molecular patterns, DAMPs (ATP, uric acid crystals, nucleic acids or inflammatory cytokines) and the amplification of inflammation (15).

Interest in epitranscriptomic regulation of bacterial infection and host immune responses has measurably increased in the last decade. Technological breakthroughs such as RNA modification mapping at high-resolution, but also the recognition that this is a comparatively underexplored area of post-transcriptional control have fueled the attention. In the sections that follow, we discuss recent advances in *Pseudomonas aeruginosa* epitranscriptome research and explore how specific RNA modifications regulate innate immune cell function.

## RNA modifications in bacterial fitness, virulence and antimicrobial resistance

### Transfer RNA modifications essential for virulence and stress responses

Transfer RNAs are indispensable for protein synthesis. They serve as adaptor molecules loaded on their 3’-CCA end with an amino acid, which they transfer to the growing polypeptide chain inside the ribosome, following codon-anticodon recognition. Transfer RNAs have a characteristic cloverleaf-like secondary structure with a few prominent features: an acceptor stem formed by the interaction of the 5’ and 3’-CCA ends, a D (dihydrouridine)- and T (TψC)-loop formed by hydrogen bonding between nucleotides in the D- and T-arms respectively, a smaller variable loop and the anticodon loop, where the conserved positions 34, 35, and 36 constitute the anticodon. The D- and T-loops fold close to each other in space to form the tRNA elbow that gives the final L-shape to the 3D structure. During the codon-anticodon interaction, positions 36 and 35 base-pair with the first and second nucleotide of the codon, respectively, following Watson-Crick base-pairing rules. Position 34 is called the “wobble position”, and can accommodate irregular, non-Watson-Crick base-pairings. This is the structural basis for the fact that many modifications of tRNAs occur at position 34 - termed wobble modifications - where they can act as regulators of codon-anticodon recognition. Immediately outside the anticodon, positions 32 and 37 are also heavily modified and are important for maintenance of the correct reading frame, ensuring accurate decoding of the genetic information. In addition, many modifications are present in the body of the tRNA where they maintain its structural stability (for example, positions 16 and 20 in the D-loop, and 54 and 55 in the T-loop). Transfer RNAs undergo a complex post-transcriptional maturation process and modifications can be introduced at its various stages. These can be either sugar or base modifications that are relatively simple, such as methylation (m^1^A, m^2^G, m^7^G, and m^5^C, etc.), thiolation (s^2^U, s^2^C), base isomerization (pseudouridine; ψ) and base reduction (dihydrouridine; D). They can also be more complex, requiring multiple modification steps (m^5^s^2^U, mcm^5^Um, and ms^2^i^6^A, etc.). It is notable that modifications located in the core region (D- and T-loop) tend to be simple, while those in the anticodon loop are more complex. It is important to keep in mind that the different aminoacyl-tRNAs are not equally sensitive to the loss of modifications at concrete positions. In addition, whether or not a specific modification is placed at a particular position depends on the structural and sequence context in which the nucleoside resides, and also on the presence/absence of other modifications in proximity or at a distance. tRNA modifications can also change dynamically and their deficiency has been associated with human diseases, collectively called RNA *modopathies*. These aspects of tRNA modification biology have been comprehensively discussed by P. Barraud and C. Tisne (18), as well as by T. Suzuki (19) and we point the readers to those excellent reviews.

Modification of tRNAs is essential for the virulence of *Pseudomonas aeruginosa*, which relies on the expression of effector molecules from its various secretion systems. One such system, Type 3 Secretion System (T3SS) is used by the bacteria to inject effector molecules directly into host cells, thereby promoting the infection process. The expression of genes belonging to the T3SS is sensitive to outside signals, such as oxidative and osmotic stress, the availability of nutrients, the presence of host cells, and the intracellular levels of the second messengers cAMP and c-diGMP. This ensures that the bacteria adapt quickly to environmental changes and the mechanisms that regulate these processes are frequently conserved across species. For example, Lin et al. have shown that MiaB, a methylthiotransferase responsible for the 2-methylthio-N6-isopentenyladenosine modification (ms^2^i^6^A_37_) at position A37 of tRNAs, regulates T3SS gene expression in *P. aeruginosa* and is required for its cytotoxicity (20). By using a *PexsCEBA-LacZ* reporter PAO1 strain, the authors found that MiaB was necessary for the expression of the *exsCEBA* operon wherein critical genes that regulate T3SS gene expression reside (20). MiaB was found to downregulate the expression of components of the LadS system (*gacA*, *rsmY* and *rsmZ*), which antagonize the RNA binding protein RsmA that is an activator of T3SS (20). The authors concluded that MiaB’s regulatory effect on T3SS was not due to its enzymatic activity, however, they were unable to investigate the functional consequences of complete ms^2^i^6^A_37_ loss because of the existence of an unidentified enzyme with redundant methylthiotransferase activity. It is possible that complete loss of the modification would exacerbate the effect on the T3SS even further, and especially under conditions of stress, however, this needs further testing. Recently, Chittrakanwong et al. investigated the phenotypic effects of TrmA loss in *Pseudomonas aeruginosa* (21). The authors identified TrmA as the enzyme responsible for the methylation of m (5)U_54_ at position U54 in the T-loop of all tRNAs. In *trmA* mutant bacteria, genes involved in the hyperosmotic salinity response, polyamine catabolism and Queuosine biosynthesis were upregulated, as were proteins involved in T3SS function (for example, export proteins PscE/F/D, exotoxins ExoT/U, the master transcriptional regulator ExsA, and others) (21). Hypomodification of m^5^U_54_ affected the abundance of certain tRNAs (some Pro and Ser tRNAs were increased, while some fMet, Ile, Arg and others were decreased) (21). The authors investigated codon usage in the proteins upregulated in *trmA* mutants and found that they were enriched for the AGU codon, recognized by tRNA^Ser^ GCU, which was increased in abundance. Conversely, the majority of downregulated proteins were enriched for the AUC codon, recognized by tRNA^Ile^ GAU, which had decreased abundance (21). Hence, this study showed that TrmA can regulate aminoacyl-tRNA abundance through m^5^U_54_, which affects the translation of transcripts sensitive to those tRNAs.

A direct role for tRNA modification in *Pseudomonas aeruginosa* pathogenicity *in vivo* was demonstrated by Srimahaeak et al. (22) They found that Glucose-inhibited division protein A (GidA) is essential for placing carboxymethyl-aminomethyl (cmnm) and methyl-aminomethyl (mnm) on uridines at the wobble position U34 of tRNAs in *Pseudomonas aeruginosa* strain PA14. The authors showed that *gidA* mutant bacteria had decreased levels of the KatA and KatB catalase enzymes, which resulted in increased bacterial susceptibility to H_2_O_2_ (22). The mutant bacteria also had reduced swimming and swarming abilities, as well as a decreased biofilm formation capability. Using macrophages or *C. elegans* as infection models, *P. aeruginosa* PA14 *gidA* mutants showed attenuated virulence *in vitro* and *in vivo* (22). More recently, Susanne Häussler’s group performed a detailed investigation of GidA and described a codon-bias mechanism dependent on tRNA modification of U_34_, whereby specific proteins important for motility and pathogenicity are efficiently translated during early stages of infection (23). Using two different *in vivo* models of infection Krueger et al. showed that, as previously observed, *gidA* mutants of *Pseudomonas aeruginosa* PA14 had severe virulence defects and were significantly less cytotoxic against macrophages infected *in vitro* (23). In addition to motility defects, the *gidA* mutants also produced less of the virulence factors pyocyanin and rhamnolipid, and formed less well organized biofilms, which were sensitive to treatment with ciprofloxacin (23). The authors confirmed that in the absence of GidA enzymatic activity, the overall levels of cmnm^5^U_34_, mnm^5^U_34_, and mnm^5^s^2^U_34_ were drastically reduced, while the level of the precursor modification s^2^U_34_ was increased.^23^ They found that specific tRNAs, namely tRNA^Gln^ UUG and tRNA^Arg^ UCU carried the mnm (5)s^2^U_34_ modification, while tRNA^Gly^ UCC was predominantly decorated with cmnm^5^U_34_ and to a lesser extent, with mnm^5^s^2^U_34_, and that these modifications were lost in *gidA* mutant bacteria. Remarkably, a reporter assay showed that codons with U-A base-pairing in the wobble position (such as AGA and UUA recognized by tRNA^Arg^ UCU and tRNA^Leu^ UAA, respectively) were translated less efficiently in *gidA* mutants.^23^ Using analysis of ribosome occupancy, the study also showed that in the absence of U34 modification, aborted translation was observed downstream of AGA codons. Interestingly, the authors found that AGA codons are rare and cluster in the beginning of certain genes in PA14 and also appear near start or stop codons. As a consequence, virulence and motility-related effector proteins, as well as master transcriptional regulators of quorum-sensing and virulence, such as RhlR and LasR were all downregulated in *gidA* mutant bacteria (23). Thus, this study showed that AGA codons, which are sensitive to U34 modification loss, can shape the cell’s gene expression program and proteome. A similar type of regulation of ribosomal pausing by tRNA hypomodification at U34 has been previously described in eukaryotic cells (24), indicating that these mechanisms are evolutionarily conserved. Finally, the authors tested 414 P*. aeruginosa* clinical isolates in early stationary phase and found an inverse relationship between *gidA* expression levels and bacterial pathogenicity. They also found that GidA expression was dynamic during infection - it is highly expressed at the early stages of an infection when it drives the expression of factors important for pathogenicity and is then downregulated in later stages in order to promote bacterial persistence (23). Next, Susanne Häussler’s group turned their attention to 5-hydroxyuridine (ho^5^U_34_) modification of tRNA and showed that it is also important for *Pseudomonas aeruginosa* pathogenicity (25). Frommeyer et al. identified TrhP/TrhO as the enzymes responsible for ho^5^U in *P. aeruginosa* PA14, which is also the precursor base modification for downstream hydroxylation derivatives (i.e. cmo^5^U, mcmo^5^U and mcmo^5^Um, collectively called xo^5^U_34_), such that TrhP/O expression influences them as well.^25^ Double *trhP/O* mutant bacteria had reduced fitness, reduced cytotoxicity against lung epithelial A549 cells *in vitro* and reduced pathogenicity against *G. mellonella* larvae infection *in vivo* compared to wild-type PA14 (25). In *trhP/O* mutants, the authors observed reduced translational efficiency of transcripts containing codons for Leu, Ser, Pro and Thr, while transcripts with codons for Val (GUU, GUA) and Ala (GCU) had a higher translation efficiency, indicating that these are all xo^5^U-dependent codons (25). However, these effects were subtle. Accordingly, Frommeyer et al. identified differentially expressed proteins in *trhP/O* mutants (24 up- and 27 downregulated), but they were unable to identify proteins whose synthesis was sensitive to hypomodified xo^5^U_34_ tRNA (25).

Resistance to oxidative stress promotes bacterial fitness and survival during infection. Thongdee et al. have reported TrmB as a m^7^G_46_ methyltransferase that positively regulates KatA and KatB expression and contributes to enhanced *Pseudomonas aeruginosa* resistance against H_2_O_2_ (26). Specifically, TrmB was shown to methylate position G46 located in the tRNA variable loop. TrmB mutant bacteria had increased sensitivity to hydrogen peroxide and, interestingly, in wild-type PA14 *trmB* expression was induced (2.6-fold) following H_2_O_2_ exposure, which resulted in increased total m^7^G_46_ tRNA methylation (26). Analysis of reporter transcripts indicated that in *trmB* mutant bacteria the translation efficiency of Phe codons (tRNA^Phe^ GAA) and Asp codons (tRNA^Asp^ GUC) was reduced (26). This is notable, because effects on translation efficiency are usually observed for modifications located in the anticodon loop. The lack of *trmB* led to decreased KatA and KatB abundance as well as to lower catalase enzymatic activity, which was especially pronounced under H_2_O_2_ stress (26). Amazingly, the Phe UUC, Asp GAC, and Asp GAU codons were found to be enriched in *katA* and *katB* transcripts in *P. aeruginosa*, compared to the complete genome (26). Thus, this study demonstrates that regulation of the expression of essential stress-response proteins through codon bias can be dictated by tRNA modifications outside the anticodon.

Recognizing the importance of tRNA modifications for different aspects of bacterial physiology has led to efforts for comprehensive tRNA modification profiling of *Pseudomonas aeruginosa* (27, 28). It should be noted, however, that in order to obtain sufficient biomass, the bacteria in both of the studies discussed below were grown in Luria-Bertani or chemically-defined media, which may affect tRNA modification type and placement. Nevertheless, Mandler et al. mapped tRNA modifications at nucleotide resolution and used the well-characterized bacterium *E. coli* as a reference to predict modifications that are conserved between the two species (27). This comparison showed that many modifications at positions 32 and 34 (for instance, Cm_32_ on tRNA^Thr^ UCU and Q_34_ on tRNA^Asn^ GUU), were conserved, including all at position 37: m^2^A, m^1^G, ms^2^i^6^A, cyclic N6-threonylcarbamoyl adenosine (ct^6^A) and N6-methyl-N6-threonylcarbamoyl adenosine (m^6^t^6^A). They also identified modifications unique to *P. aeruginosa*, such as N3-(3-amino-3-carboxypropyl)-uridine at position U46 (acp^3^U_46_) on tRNA^Gln^ UUG within the variable loop. They were also able to identify the enzymatic activity TapT responsible for it (27). It will take further investigation to systematically characterize *P. aeruginosa* mutants for these modifications and it will be interesting to see whether or not modifications that are conserved across multiple Gram-negative bacteria have similar phenotypes, and conversely whether unique modifications are inherently important for the particular species. In their insightful breakthrough study, Sun et al. undertook a high-throughput tRNA modification mapping effort based on LC-MS/MS and leveraged a 5764-strain non-redundant library (29) of *Pseudomonas aeruginosa* PA14 transposon insertion mutants (28). They identified 35 tRNA modifications in wild-type *P. aeruginosa* PA14, in addition to 6 biosynthetic intermediates (s^2^U, nm^5^s^2^U, cmnm^5^s^2^U, mo^5^U, preQ1 and oQ) for a total of 41 tRNA modifications (28). When interrogating the mutant library, the authors identified 313 genes, whose mutational inactivation caused significant changes in 30 modifications. In addition to confirming known homologs of modification enzymes, Sun et al. identified new enzymatic activities and their respective modifications: TrmS as responsible for Gm at position 18, TrmV (m^2^A at position 37), CsdA (t^6^A, ct^6^A and m^6^t^6^A at position 37), and RlmF (m^6^A at position 37; identified as a dual-specificity methyltransferase for rRNA and tRNA) (28). The authors also confirmed the previous finding that TapT is responsible for acp^3^U at position 46/47. Importantly, they showed that mutational inactivation of a single enzyme in the beginning of a biochemical pathway can affect a group of modifications downstream (for example, *miaA* loss affected i^6^A, io^6^A, ms^2^i^6^A, and ms^2^io^6^A levels at position 37) (28). Furthermore, mutations in enzymes for the synthesis of cofactors used by tRNA modifying enzymes were also shown to have profound effects (for example, loss of the SAH-hydrolase *sahH* affects all modifications which require SAM as a methyl group donor) (28). Finally, mutations in genes important for seemingly unrelated functions, such as translocation and biogenesis of cobalamin, affected QueG-mediated conversion of epoxyqueuosine (oQ) to queuosine (Q), which uses cobalamin (28). This is clear evidence that intracellular biochemical pathways, metabolic activity and RNA modifications are interconnected. Coming back full circle, the authors also demonstrated that the activity of other enzymes within its extended network can alter MiaB activity, and hence, the levels of the modifications i^6^A_37_ and ms^2^i^6^A_37_. Amazingly, this network was shown to contain 104 genes important for bacterial functions ranging from iron-sulfur cluster assembly and biogenesis, to NO detoxification and oxidative stress response (28). This suggests that certain tRNA modification enzymes can serve as regulatory hubs by integrating signals from diverse cell activities to generate protein translation and gene expression outcomes.

### tRNA and rRNA modifications involved in antimicrobial resistance

Antimicrobial resistance acquired by *Pseudomonas aeruginosa* following tRNA modification dysregulation may be an indirect effect of compensatory mechanisms induced as stress-responses. For example, MexC and MexD were among the proteins upregulated in *trhP/O* mutant bacteria (from Frommeyer et al. (25*)* discussed above). These proteins belong to the MexCD-OprJ efflux pump system that is upregulated in the presence of antibiotics and contributes to resistance against xenobiotics. However, the upregulation of genes from the *mexCD-oprJ* operon in *trhP/O* mutants was considered to be secondary to the accumulation of chorismate in these bacteria and its redirection to alternative biochemical pathways, such as the increased synthesis of pyocyanin, instead of being directly caused by xo^5^U_34_ hypomodification (25). In addition, despite its ubiquitous nature, the m^5^U_54_ modification (discussed in Chittrakanwong et al. (21*)* above) did not appear to have any appreciable effect on bacterial motility or biofilm formation. After testing 236 different compounds, remarkably, *trmA* mutant *Pseudomonas aeruginosa* PA14 only showed increased resistance to polymyxin B. Unexpectedly, the resistance was not associated with changes in the cell membrane structure, but rather to lower intracellular ROS levels after polymyxin B exposure of *trmA* mutants compared to wild-type PA14. However, the authors did not investigate whether or not this was a direct effect of m^5^U_54_ hypomodification (21).

Many antibiotics commonly used in the clinic bind to the 16S rRNA or the 23S rRNA of the 30S and 50S ribosomal subunits, respectively, leading to inhibition of protein translation. The sequence differences between prokaryotic and eukaryotic rRNA are sufficient enough that the antibiotics act selectively on bacterial ribosomes. Until recently, 16S and 23S rRNAs were thought to be conserved across bacterial species. However, Ekemezie et al. have demonstrated substantial natural sequence variation, even among closely related bacteria, that can result in intrinsic antimicrobial resistance (30). Their study showed that *Pseudomonas aeruginosa* has variable drug-binding residues in 23S rRNA (Cs in positions 2533/2534), but they were outside of positions frequently mutated in antibiotic-resistant bacteria (30). In addition to natural variation and acquired mutations of rRNA, modifications are another way bacteria can control resistance to antimicrobials. Bacterial rRNA is extensively modified, however in contrast to the diversity observed in tRNAs, the overwhelming majority of modifications are isomerization of uridine (pseudouridine; ψ) and 2’-O-methylation of the ribose (Nm), and only a small fraction are base modifications (such as m^6^A, m^5^C, and m^7^G). Phatinowat et al. investigated the effect of KsgA mutational inactivation in *P. aeruginosa* PA14 (31). KsgA is a 16S dimethyltransferase responsible for the N6-N6-dimethyladenosine modification (m^6,6^A) of positions A1518/1519, which had previously been shown to be important for ribosome biogenesis and protein translation. The authors found that in *ksgA* mutant bacteria the modification in this position is lost (31). Using Biolog phenotypic microarrays, they found that *ksgA* mutants have sensitivity towards three antimicrobial agents, namely menadione, tylosin and hygromycin B. Mechanistically, the sensitivity to menadione was explained by reduced SodM protein abundance leading to reduced superoxide dismutase enzymatic activity in *ksgA* mutant bacteria compared to the wild-type (31). The basis for tylosin sensitivity was suggested to be the subtle downregulation of tylosin-resistance genes, such as elongation factors EF-Tu and EF-Ts (*tufA*, *tufB* and *tsf*) in *ksgA* mutants (31). This finding indicates that rRNA modifying enzymes can alter - whether directly through rRNA hypomodification, or indirectly is unknown - the expression of elongation factors which transport aminoacyl-tRNAs to the ribosome, and is especially intriguing since EF-Tu has recently been suggested by Moustafa et al. (32) as a vaccine candidate for *Pseudomonas aeruginosa*. The study also found that, as reported previously for other bacteria, *ksgA* mutant *P. aeruginosa* PA14 had increased resistance to kasugamycin, which is likely due to disruption of antibiotic-ribosome binding (31).

The feasibility of targeting rRNA methyltransferases with inhibitors in order to overcome pan-aminoglycoside resistance has recently been tested by Dey et al. (33) They developed a number of small-molecule inhibitors of NpmA, a resistance-associated methyltransferase responsible for the N1-methyladenosine modification at position 1408 (m^1^A_1408_) in the 16S rRNA. NpmA belongs to a family of enzymes expressed in clinically important pathogens, including *Pseudomonas aeruginosa*. The compounds had IC_50_ = 68 µM to IC_50_ = 99 µM and resulted in less than 25% residual enzymatic activity (33). Whether or not they, or other similar compounds, can be effective in overcoming antibiotic resistance during infection remains to be seen.

### Bacterial ncRNA modifications

In addition to T3SS, *Pseudomonas aeruginosa* possess T6SS, a bacteriophage tail-like system that delivers toxic effecter molecules into other prokaryotic rivals or eukaryotic cells. In an elegant study, Bullen et al. identified RhsP2 as a toxin that is delivered through H2-T6SS into competing bacterial cells, eliciting potent antibacterial effects (34). RhsP2 exhibited promiscuous activity and catalyzed 2’-O-ADP-ribosylation of many ncRNAs and all tRNAs within the targeted cells, while rRNAs were mostly protected inside the risbosome (34). RhsP2 was shown to structurally resemble protein-targeting ADP-ribosyltransferases, yet it had no protein modification activity. The authors found that Rhsp2 did not depend on sequence specificity, but instead relied on dsRNA structures within its substrates, explaining its promiscuity (34). In the targeted cells, ADP-ribosylated tRNAs were unable to undergo aminoacylation leading to complete disruption of protein synthesis. In addition, ncRNAs such as transfer-messenger RNA (tmRNA), the small regulatory RNA CsrB, the 4.5S RNA and RNase P were also modified (34). Their modification inhibited diverse processes related to protein secretion, recycling stalled ribosomes, and processing of tRNA transcripts, ultimately resulting in cell death (34).

The molecular targets and functional outcomes of RNA modification in *P. aeruginosa* discussed above have been summarized in **Table 2**.

**Table 2:** Molecular targets and functional outcomes of RNA modification in *P. aeruginosa* cells.

Enzyme/modification	Molecular target	Functional outcome/phenotype [ref.]
tRNA modification		
MiaB/ms^2^i^6^A_37_	Targets tRNAs recognizing codons beginning with U.	Reduces gacA, rsmY, and rsmZ expression; Necessary for activation of T3SS expression from *exsCEBA*; Required for bacterial cytotoxicity. (20)
TrmA/m^5^U_54_	Targets all tRNAs. Hypomodification results in altered tRNA abundance.	Affects the translation of transcripts sensitive to specific tRNAs, such as for the T3SS proteins PscE/F/D, exotoxins ExoT/U, and ExsA. (21)
TrmB/m^7^G_46_	Hypomodification lowers the translation efficiency of tRNA^Phe^ GAA and tRNA^Asp^ GUC codons.	Positive regulator of KatA and KatB expression; Enhanced resistance to H_2_O_2._ (26)
GidA/cmnm^5^U_34_, mnm^5^U_34_ and mnm^5^s^2^U_34_	Targets tRNA^Gln^ UUG, tRNA^Arg^ UCU and tRNA^Gly^ UCC. Hypomodification results in aborted translation.	Loss results in increased susceptibility to H_2_O_2_ due to decreased KatA and KatB; Affects motility, virulence factor production, quorum-sensing and biofilm formation. (22, 23)
TrhP and TrhO/xo^5^U_34_	Hypomodification results in: ↑translation efficiency for transcripts with Val (GUU, GUA) and Ala (GCU); ↓translation efficiency for transcripts containing Leu, Ser, Pro and Thr codons.	Subtle effects on protein levels. (25)
TapT/acp^3^U_46_	tRNA^Gln^ UUG	unknown. (27, 28)
rRNA modification		
KsgA/m^6,6^A	positions A1518/1519 of 16S rRNA	Mutant bacteria have increased sensitivity towards menadione, tylosin, hygromycin B, and kasugamycin (31).
ncRNA modification		
RhsP2/2’-O-ADP-ribosylation	Targets many ncRNAs (tmRNA, CsrB, 4.5S RNA, RNase P)	Antibacterial effect; Inhibits translation, protein secretion and processing of tRNA transcripts in order to eliminate rival bacteria (34).

During infection, the examination of RNA modification dynamics in bacterial cells alone provides a one-sided perspective on the interaction between the microbe and the host. A systems-level understanding of their bi-directional crosstalk requires examination of epitranscriptomic changes that also occur in the host. Such changes govern tissue-resident and infiltrating innate immune cell function and activation, as well as the structural cell response to infection (e.g. by epithelial and endothelial cells). We discuss these host-side dynamics in the next section.

## RNA modifications in host responses to infection

### M6A - a major multi-functional internal mRNA modification

RNA modifications, such as N6-methyladenosine (m6A) play a role in diverse immunity-related physiological processes such as hematopoietic stem cell differentiation and immune cell homeostasis. During infection they are also involved in regulating intrinsic immune cell functions, such as cell activation, mobilization and chemotaxis, secretion of inflammatory mediators, and antigen-presentation. M6A is the most abundant and well-studied internal mRNA modification in eukaryotes. It is placed co-transcriptionally on transcripts by a large methyltransferase complex containing the core proteins METTL3/METTL14/WTAP, with METTL3 responsible for the catalytic activity. The way m6A is interpreted by the cell and its functional consequences depend on recognition by YTH-, hnRNP- and IGF2BP-families of m6A reader proteins. The m6A modification can also be actively removed by the FTO and ALKBH5 demethylases. Most of the research on m6A dynamics and regulation in infection has been focused on viral disease and we have recently reviewed many of these studies (please see Leseva et al. (35) and references therein). In this section we focus on its regulation of innate immune cell functions during bacterial infection and sepsis.

### M6A as a regulator of innate immune cell function

**Figure 3:**
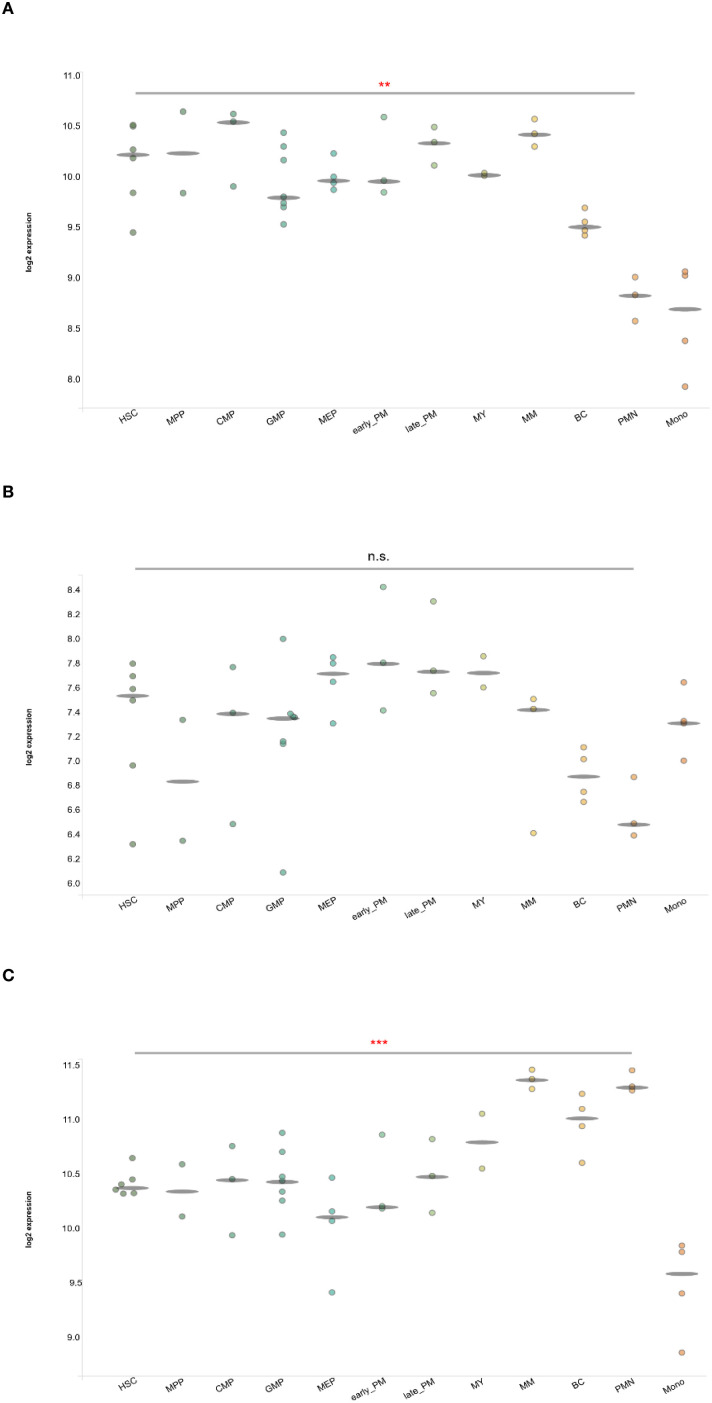
Expression of METTL3 **(A)**, METTL14 **(B)** and WTAP **(C)** during normal human hematopoiesis. Microarray data is from Rapin et al. (105) and Svendsen et al. (106) [GEO accession number GSE42519]. The data is plotted in Bloodspot (107) (bloodspot.eu). Abbreviations and immunophenotypes: *HSC-Hematopoietic stem cell* (Lin- CD34+ CD38- CD90+ CD45RA-); *MPP-Multi-potential progenitors* (Lin- CD34+ CD38- CD90- CD45RA-); *CMP-Common myeloid progenitor* (Lin- CD34+ CD38+ CD45RA- CD123+); *GMP-Granulocyte monocyte progenitors* (Lin- CD34+ CD38+ CD45RA+ CD123+); *MEP-Megakaryocyte-erythroid progenitor* (Lin- CD34+ CD38+ CD45RA- CD123-); *early_PM-Early Promyelocyte* (Lin- FSChi SSCint CD34- CD15int CD49dhi CD33hi CD11b- CD16-); *late_PM-Late Promyelocyte* (Lin- FSChi SSChi CD34- CD15hi CD49dhi CD33hi CD11b- CD16-); *MY-Myelocyte* (Lin- FSChi SSChi CD34- CD15hi CD49dhi CD33hi CD11bhi CD16-); *MM-Metamyelocytes* (Lin- FSChi SSChi CD34- CD15hi CD49d- CD33- CD11bhi CD16-); *BC-Band cell* (Lin- FSChi SSChi CD34- CD15hi CD49d- CD33- CD11bhi CD16int); *PMN-Polymorphonuclear cells* (Lin- FSChi SSChi CD34- CD15hi CD49d- CD33- CD11bhi CD16hi); *Mono-Monocytes* (CD14+ CD16-); Comparisons between HSC and PMN T-test- n.s., non-significant; **p<0.01; ***p<0.001.

Adult hematopoietic stem cell differentiation is a regulated process of cell differentiation in the bone marrow during which hematopoietic stem cells (HSC) differentiate into multipotent progenitors (MPP) and progressively commit to different immune cell lineages in order to generate mature immune cells. We have previously reviewed the dynamics of m6A during this process (please see Leseva et al. (35*)* and references therein). Here, we reiterate that during normal mouse (36) and human myelopoiesis, *METTL3*/*14* levels decline (see **Figure 3**). This is significant as it results in low background m6A levels in the major innate immune cells of the myeloid lineage during homeostasis (i.e. in monocytes, neutrophils, macrophages and dendritic cells). **Figure 3** shows the downregulation of *METTL3* expression in human polymorphonuclear cells (PMN) and monocytes (MONO) compared to HSC, MPP and other progenitor cells (**Figure 3A**). This suggests that the upregulation of METTL3 and consequent increase of m6A following immune stimulation, such as by infection, can facilitate a sensitive, dynamic and robust regulatory response, especially if other core proteins of the methyltransferase complex are highly co-expressed (see METTL14 and WTAP expression levels in metamyelocyte-MM, band cell-BC and PMN cells, **Figure 3B, C**).

Indeed, *Mettl3* expression is induced following stimulation and is required for the activation of various innate immune cell types (37–39). For example in LPS-induced endotoxemia and acute lung injury in mice, METTL3 is required for the efficient release of neutrophils from the bone marrow into the circulation (37). Luo et al. showed that following LPS treatment, TLR4 signaling is inhibited in conditional knockout Lyzm-Cre mice in which *Mettl3* is not expressed in the neutrophils and macrophages (37). METTL3 was shown to target *Tlr4* for m6A modification, which had a positive effect on transcript stability and translation efficiency. By enhancing neutrophil TLR4 signaling, METTL3 acted to increase surface expression of CXCR2 in response to CXCL1/2, which facilitates the mobilization of bone marrow neutrophils to the periphery (i.e. to the blood and tissues) following stimulation (37). Professional antigen presenting cells, such as dendritic cells and macrophages, are required to stimulate an adaptive immune response for pathogen clearance. Wang et al. have shown that Mettl3 is required for dendritic cell (DC) activation and function (38). *Mettl3*, *Mettl14* and *Wtap* expression, as well as m6A levels increase in LPS-treated mature DCs, compared to immature dendritic cells. Methylated RNA immunoprecipitation (RIP)-seq analysis revealed that new m6A peaks in mature DC cells were enriched in transcripts belonging to the NLR- and TNF-signaling pathways compared to immature cells, and for NF-κB signaling when compared to regulatory DC cells (38). This indicates that m6A upregulation following stimulation has functional consequences. Mature *Mettl3* knockout dendritic cells express lower levels of co-stimulatory molecules such as CD86, CD80, and CD40, and respond with low IL-6, IL-12 and TNF-α levels following LPS stimulation (38). Further, METTL3-mediated m6A modification of *Tirap*, *CD80* and *CD40* led to their increased translation (38). TIRAP is an adaptor protein for TLR2 and TLR4, and is essential for downstream NF-κB signaling and pro-inflammatory cytokine production (see **Figure 2**). Thus, m6A reinforces NF-κB signaling in DC cells. METTL3 and m6A have also been shown to be important for macrophage activation and function. Tong et al. have shown that METTL3 directly targets *Irakm* mRNA for m6A modification, which results in its efficient degradation. If this process is perturbed, IRAKM is accumulated in macrophages, which results in inhibition of TLR4 signaling in response to stimulation (39). Indeed, conditional *Mettl3*-knockout Lyzm-Cre mice were more susceptible to infection and had higher bacterial loads (39). In a mouse model of LPS-induced acute lung injury, Cao et al. observed that the expression of METTL14 was increased at both the gene and protein level in monocyte-derived macrophages recruited to the inflamed ALI lungs, which also led to an increase in their overall m6A levels (40). They also observed an upregulation of METTL14 in LPS-treated macrophages, which led to the activation of the NLRP3 inflammasome. Mechanistically, this was achieved through targeting of *NLRP3* mRNA for m6A modification and its consequent stabilization facilitated by binding to IGF2BP2 (40). Thus, the METTL14-IGF2BP2 axis might contribute to the hyperactivation of the NLRP3 inflammasome during ALI and acute respiratory distress syndrome (ARDS). NLRP3 inflammasome hyperactivation leads to secretion of massive amounts of mature IL-1β (**Figure 2**) and IL-18, which can trigger alveolar cell pyroptosis during inflammatory lung injury.

### M6A in sepsis-associated lung pathology

Sepsis is a life-threatening organ dysfunction caused by a dysregulated systemic host response to infection and is associated with elevated serum lactate levels, which are reflective of cellular dysfunction (41). During infection the acute demand for myeloid cells, the first responders to invading pathogens, triggers emergency myelopoiesis (please see review by Swann et al. (42)). It had been previously shown that in critically ill patients with sepsis caused by Gram-negative bacteria, leukocytes had reduced ALKBH5 expression levels (43). Using a mouse model of polymicrobial sepsis (cecal ligation and puncture; CLP), Liu et al. have begun to clarify the critical role of m6A in emergency granulopoiesis (i.e. the generation of neutrophils) (44). They observed that knockout mice for the m6A demethylase *Alkbh5* had differentially expressed genes involved in neutrophil proliferation and differentiation (for example, downregulation of *Ly6g*, *Itgam*, *Csf3r* and others) (44). This was also recapitulated *in vitro* in human ALKBH5 KO neutrophils. Mechanistically, ALKBH5 was shown to demethylate *CSF3R* mRNA which leads to its increased stability and promotes the intracellular and surface expression of the receptor G-CSF3R (44). This receptor binds to granulocyte colony-stimulating factor (G-CSF) and triggers downstream STAT3 signaling to induce emergency immature neutrophil expansion and mobilization from the bone marrow. Thus, the downregulation of ALKBH5 previously observed in sepsis patients might result in impeded emergency granulopoiesis and antibacterial innate immune defense.

Hyperlactatemia (defined as serum lactate >2 mmol/L) is a predictive biomarker for septic shock, a subset of sepsis, and is positively associated with higher mortality rates because of the underlying extensive cellular metabolic abnormalities (41). Intracellular lactate in the form of lactyl-CoA is a substrate for histone lactylation, which directly connects cell metabolism with epigenetic regulation of gene expression (45). Di et al. have shown that H3K18la and H3K18la/ac levels are elevated in peripheral blood mononuclear cells (PBMCs) from critically ill ICU patients with sepsis or septic shock compared to non-infectious patients, and positively correlate with serum lactate levels (46). They observed a modest, but statistically significant positive correlation between H3K18la/ac ratios and Procalcitonin levels (PCT) (Spearman ρ=0.462, P<0.001). The authors also showed that H3K18la/ac can be used as a biomarker to identify, among all ICU patients, those that were critically ill with infectious disease, as well as the septic shock subset of patients (46). As a biomarker, the H3K18la/ac ratios had statistical power comparable to that of PCT and were shown to be positively associated with disease severity [measured using Acute Physiology and Chronic Health Evaluation (APACHE II) and Sequential Organ Failure Assessment (SOFA) scores], duration of stay in the ICU and mechanical ventilation time (46). The H3K18la/ac ratios also had a small but significant positive correlation with serum levels of the immunosuppressive interleukin IL-10 (Spearman ρ=0.272, P = 0.007), and a stronger negative correlation with the protective interleukin IL-5 (47) (Spearman ρ=-0.342, P<0.001) (46). These observations might help explain the long-term immunosuppression observed in patients who have recovered from severe sepsis (H3K18la will be discussed again later in the section on innate inflammatory memory).

Cellular metabolic dysfunction resulting in increased lactate levels is not unique to sepsis. Recently, Xiong et al. have reported that in the context of colon cancer, accumulation of lactate in the tumor microenvironment leads to the induction of *Mettl3* expression in tumor-infiltrating myeloid cells through increased H3K18la of its promoter region (48). This is notable, because it demonstrates that *Mettl3* gene expression is sensitive to intracellular lactate. In addition, the authors found that the METTL3 protein itself can undergo lactylation at positions K281 and K345, which result in a stronger ability to bind its target RNAs without affecting its m6A enzymatic activity. Lysine lactylation of other m6A regulators have also been reported. For example, ALKBH5 was shown to be lactylated at position K284, which improved its interaction with *IFN-β* mRNA and enhanced innate antiviral responses (49). It is tempting to speculate that a similar mechanism of lactate-induced METTL3 expression and lactyl-PTM of m6A regulatory proteins may be at play during sepsis. For example, Wu et al. have reported that in a mouse model of CLP-induced sepsis, alveolar epithelial cell *Mettl3* and m6A levels are increased through the same H3K18la-mediated process described above (50). Mechanistically, the authors found that METTL3 targets acyl-CoA synthetase long-chain family member 4 (*ACSL4*) for m6A modification, which stabilized the transcript through binding by YTHDC1 (50). Increased intracellular lactate levels and ACSL4 led to mitochondrial activation and the accumulation of reactive oxygen species (ROS). This resulted in ferroptosis, a type of regulated cell death involving iron-dependent lipid peroxidation (50). Thus, METTL3 can be used as a target to limit alveolar cell death during sepsis-induced acute lung injury, which the authors demonstrated with the METTL3-specific inhibitor STM2457. Intraperitoneal injection of STM2457 prolonged the survival of the septic mice, and alleviated their lung edema and inflammation (50). However, using a similar sepsis model, Chen et al. have shown that m6A, *Mettl3* and *Mettl14* are downregulated in the lung endothelium, which exacerbated damage to the endothelial barrier (51). They proposed that METTL3 can inhibit endothelial injury observed during sepsis-induced ARDS by modifying *Trim59*, which leads to its increased stability through binding to YTHDF1 and downstream inactivation of the NF-κB signaling pathway (51). The discrepancy between the studies pertaining to the levels of m6A and Mettl3 might be due to the different cell types that were investigated (alveolar epithelial vs. lung endothelial cells) as well as the severity of the lung injury (ALI vs. ARDS). However, these results underscore that while the METTL3-m6A axis might be damage-inducing in one cell type of the tissue, it could be protective in another, which complicates its use as a therapeutic target. The role of host RNA modifications in sepsis pathobiology has only recently begun to be considered and much more research will be required to accumulate high-confidence data. Most of the available literature concerning RNA modifications in sepsis relates to m6A and has been reviewed by Zhang et al. (52).

### Non-m6A RNA modifications involved in *Pseudomonas aeruginosa* infection and sepsis

Compared to N6-methyladenosine, much less is known about the role of other internal mRNA modifications during bacterial infection and in septic lung injury. Recently, Huang et al. showed that in *Pseudomonas aeruginosa*-induced acute lung injury, acetylation of *Hmgb1* mRNA by the acetyltransferase NAT10 exacerbates lung damage by inducing mitochondrial dysfunction in the lung epithelial cells (53). NAT10 catalyzes the N4-acetylcytidine modification (ac4C) and when present within coding sequences promotes mRNA stability and protein translation (54). Using a mouse model of *P. aeruginosa* PAO1 infection, Huang et al. observed that NAT10 is upregulated in the lung tissue following infection (53). Knockdown of NAT10 reduced the lung tissue damage and pulmonary microvascular permeability, as well as the lung bacterial load in infected mice. In addition, it also reduced the number of broncho-alveolar lavage fluid (BALF) neutrophils and the levels of the cytokines KC, IL-6 and TNF-α in BALF, suggesting that induction of NAT10 during infection promotes the lung pathology, as well as the recruitment of neutrophils and local inflammatory signaling (53). Silencing of NAT10 resulted in reduced apoptosis in mouse epithelial cells *in vitro*, thereby promoting cell viability. Importantly, silencing of NAT10 decreased the oxidative stress induced by the infection, measured by reduced intracellular ROS and sustained mitochondrial membrane potential, mtDNA copy number and ATP production (53). Mechanistically, the authors found that NAT10 can target the CDS of the *Hmgb1* transcript for acetylation, which results in its increased stability and up-regulated HMGB1 protein level (53). In addition, the positive effects of NAT10 silencing on cell viability and oxidative stress following the infection could be overcome by HMGB1 overexpression both *in vitro* and *in vivo*, establishing the NAT10-ac4C-HMGB1 regulatory axis as an adverse pathology-promoting pathway during *P. aeruginosa* lung infection (53).

N4-acetylcytidine modification of mRNA has also recently been shown to regulate neutrophil pyroptosis and lung endothelial cell injury during sepsis (55, 56). Zhang et al. reported that NAT10 expression at the mRNA and protein levels is decreased in BALF neutrophils isolated from patients with sepsis compared to healthy controls, and is inversely correlated with disease severity (R=-0.499, P = 0.0251) (55). Using the CLP-induced mouse model of sepsis and a secondary infection with *Pseudomonas aeruginosa*, the authors observed that NAT10 is similarly downregulated in blood, spleen and BALF neutrophils, as well as in the lung tissue during sepsis in mice. This resulted in an overall decreased neutrophil ac4C level in septic mice compared to controls (55). Neutrophil-specific overexpression of NAT10 using Mrp8-Cre mice improved survival following induction of sepsis, alleviated sepsis-induced lung injury and decreased neutrophil cell death through pyroptosis (55). By analyzing differentially expressed genes upregulated in NAT10 overexpressing neutrophils, which overlap with genes downregulated in CLP-induced sepsis, Zhang et al. identified the UNC-51 like kinase (*Ulk1*) mRNA as a NAT10 target for CDS ac4C modification (55). ULK1 is known to phosphorylate the intracellular adaptor molecule STING and to act as a negative regulator preventing excessive Type I interferon production through the STING/IRF3 pathway (57). Indeed, Zhang et al. found that neutrophil-specific overexpression of NAT10 inhibits the activation of STING through the ac4C modification and stabilization of *Ulk1* mRNA (55). Interestingly, ULK1 was also downregulated in BALF neutrophils from patients with sepsis, indicating that the NAT10-ULK1-STING regulatory axis is conserved in sepsis and its activation may be beneficial (55). In their study, Xing et al. found that NAT10 expression was increased *in vivo* in the lung tissue of patients with ARDS, as well as *in vitro* in LPS-treated microvascular endothelial cells (56). Knockdown of NAT10 increased the viability of the endothelial cells potentially through the inhibition of ferroptotic programmed cell death, confirming the finding by Huang et al. that NAT10 plays a negative role in promoting respiratory disease (56). In a CLP model of sepsis in rats, NAT10 knockdown was shown to alleviate the lung tissue injury. NAT10 targeted the transferrin receptor *Tfrc* mRNA for ac4C modification, which stabilized it in HULEC-5a endothelial cells (56). This established the adverse role of the NAT10-ac4C-TFRC regulatory axis. Indeed, overexpression of TFRC in HULEC-5a cells could recapitulate the negative effect of NAT10 on cell viability and induction of ferroptosis (56).

The conflicting results pertaining to the expression of NAT10 in the lung tissue, whether increased or decreased, during severe lung infection may reflect different pathogenic mechanisms of CLP-induced sepsis and direct *P. aeruginosa* inoculation in mice. They may also be due to different stages/severity of lung disease in the sepsis patients and/or the involvement of various microbial pathogens, important confounders which these studies did not report in detail. Another complicating factor for clarifying the role of NAT10 and ac4C in infectious disease is that this modification has location-specific effects. As mentioned above, ac4C in the CDS enhances transcript stability and promotes translation elongation. However, Arango et al. recently showed that 5’UTR ac4C represses translation initiation at adjacent canonical start codons and promotes non-canonical upstream initiation at less optimal sites, thereby reshaping the cellular proteome (58). In addition, transcripts coding for proteins with important functions such as cell-cycle and transcriptional regulation appear to be modified with 5’UTR ac4C more frequently, potentially making them more sensitive to NAT10 activity modulation (58). This suggests that therapeutic targeting of NAT10 may have unexpected side effects.

The molecular targets and functional outcomes of RNA modification in the mammalian host cells discussed above have been summarized in **Table 3**.

**Table 3:** Molecular targets and functional outcomes of RNA modification in mammalian host cells.

Enzyme/modification	Molecular target	Functional outcome/phenotype [ref.]
mRNA modification		
METTL3/m6A	Tlr4	Target transcript stabilization; enhanced TLR4 signaling in neutrophils; facilitated mobilization of BM neutrophils (37).
METTL3/m6A	Tirap, CD80, CD40	Enhanced translation efficiency of the targets and reinforced NF-κB signaling in dendritic cells (38).
METTL3/m6A	Irakm	Promoted target mRNA degradation and efficient TLR4 signaling in macrophages (39).
METTL3/m6A	Acsl4	Target transcript stabilization; mitochondrial activation and increased ROS; induction of ferroptosis in alveolar epithelial cells (50).
METTL3/m6A	Trim59	Target transcript stabilization; inactivation of NF-κB signaling in endothelial cells (51).
METTL14/m6A	Nlrp3	Target transcript stabilization and NLRP3 inflammasome hyperactivation in monocyte-derived macrophages (40).
ALKBH5/removes m6A	Csf3r	Target transcript stabilization promotes the expression of G-CSF3R; activated STAT3 signaling for emergency granulopoiesis (44).
NAT10/ac4C	Hmgb1 (CDS region)	Target transcript stabilization and HMGB1 upregulation; induced mitochondrial dysfunction in lung epithelial cells (53).
NAT10/ac4C	Ulk1 (CDS region)	Target transcript stabilization; inhibited activation of STING in neutrophils (55).
NAT10/ac4C	Tfrc (CDS region)	Target transcript stabilization; promoted ferroptosis in microvascular endothelial cells (56)

Infectious disease modelling has historically been done using animal models. This typically involves intranasal or intra-tracheal microbial inoculation of small laboratory animals (e.g. mice, rats and guinea pigs), and much of our understanding of acute infection as well as mammalian innate and adaptive immunity have come from them. While transgenic animals provide valuable insight into the role of specific genes in infectious disease pathology, animal models do not adequately recapitulate the complexity of disease progression, the breadth of immune responses, and the mechanisms of infection chronification observed in humans. This is due to: i) differences in anatomy and physiology; ii) human population-level genetic variation; iii) the frequent presence of comorbidities in patients; and iv) the possibility of multi-pathogen co-infection at the individual level. Laboratory animal experiments should be rigorously designed to comply with ARRIVE 2.0 (Animal Research: Reporting *In Vivo* Experiments) (59) and 3Rs guidelines (reduce, replace, refine) to ensure *in vivo* data reproducibility and protect animal welfare. However, evolving ethical considerations and international legislation (e.g. Directive 2010/63/EU of the European Parliament) are encouraging the move away from animal experimentation, whenever possible. We highlight advanced human *in vitro* models that can facilitate the investigation of epitranscriptomic regulation of host-pathogen interactions in the next section.

## Physiologically relevant *in vitro* models of *Pseudomonas aeruginosa* respiratory infection

### Ex vivo tissue-based models

*Ex vivo* recapitulation of native lung architecture and cell composition, including the presence of both structural cells and tissue-resident immune cells such as macrophages, monocytes, and T-cells, can be achieved with precision-cut lung slices (PCLS), which have been used as an organotypic model in infectious disease studies (reviewed by Viana et al. (60*)*). PCLS are generated from whole animal or human lungs (live or cadaver-donated) or individual lobes infused with a low-melting agarose-medium solution. After solidification, the tissue is cut with a vibratome or tissue slicer to uniform 150-500 µm slices (up to 1000 µm thick slices can be generated from human tissue) (60). The PCLS are maintained submerged in cell culture medium or in dynamic culture conditions and can remain viable for 7–10 days. This period is a wide enough window to investigate pathogen invasion, biofilm formation, tissue-specific innate immune responses (notably, without interference from infiltrating immune cells) and chemotherapeutic agents that attenuate or augment these processes. Because they are suitable for deep tissue, high-resolution live-cell imaging (61), and preserve cell-cell interactions and cellular metabolic activity, PCLS can be used to study host cell activation, migration, immunometabolism, and death in response to infection. Now that isolation of high-quality RNA for downstream NGS sequencing of PCLS from different species has been reported by Stegmayr et al. (62*)*, we should be able to also investigate epitranscriptomic dynamics associated with all of these processes. Establishing diseased-PCLS from patients with cystic fibrosis or COPD can be even more informative considering the fact that people with these chronic diseases are highly susceptible to respiratory infections. Recently, two studies on *Pseudomonas aeruginosa* have been reported, which used murine PCLS and the PAO1 bacterial strain in addition to clinical isolates from CF patients. In their study, Kolbe et al. (63*)* showed that only live and not heat-killed bacteria trigger an early pro-inflammatory cytokine response to infection from the PCLS cells, demonstrated by induced *Kc, Il-6 and Mip-2* expression, and that the *P. aeruginosa* flagellar protein FliC and the PscF protein of the T3SS are required to distinguish live from dead bacteria. The study also showed that interference with bacterial internalization through inhibition of actin polymerization reduced the immune response, and that the CD200R1 and MARCO receptors, expressed by professional phagocytic cells such as alveolar macrophages, function redundantly to control bacterial uptake and initiate an efficient inflammatory response. In their study, Sommer et al. (64*)* addressed the question of age-dependent changes in the immune response to bacterial pneumonia, also known as inflammaging. They showed that baseline expression levels of pro-inflammatory cytokine and chemokine genes (*Il-1β*, *Il-6*, and *Cxcl1*) and genes associated with immune cell activation, migration and phagocytosis (*Fcgr3*, *Itgam*, *Fcer1g* and others), in uninfected PCLS established from old mice are already increased and are further induced by infection (shown by secretion of TNF-α, IL-17A, CCL3). Thus, age-associated changes in the innate immune response, such as inflammaging, can be recapitulated *ex vivo* using PCLS.

### Lung-on-chip and tissue engineered human lungs

Research using human tissue is frequently hindered by issues of accessibility, which can be overcome by tissue bioengineering and the establishment of biomimetic microfluidic culture devices called Organs-on-chips (OoCs). OoCs successfully balance the sacrifice of native tissue complexity and cellular heterogeneity with the achievement of near-physiological cell-cell interactions, spatial organization and structural tissue-tissue interfaces, controlled physicochemical microenvironments and mechanical forces that all contribute to organ-level functionality. Human lung-on-chip platforms (reviewed by Bai and Ingber (65)) include alveolus-on-chip and airway-on-chip, which can be designed as two- or three-channeled setups. The channels are separated by a permeable membrane coated with extracellular matrix proteins (fibronectin, laminin, and collagen I) and lined on the one side by *in vitro* differentiated alveolar or bronchial epithelial cells, and by pulmonary microvascular endothelial cells on the other, mimicking the blood vessel interface where gas exchange occurs. The third channel can include a stromal cell interface with lung fibroblasts or smooth muscle cells. Perfusion of cell culture medium at physiological flow rates and the application of stretching recreate the mechanical stress associated with blood flow and breathing motions. Lung-on-chips have increasingly been used to investigate host-pathogen interactions, and recently a more complex immune-competent model of the human small airways was reported (66), which included immune cells, such as airway and interstitial macrophages, dendritic cells, neutrophils, NK, T- and B-cells. This model was used to recapitulate in a controlled *in vitro* environment aspects of severe H1N1 influenza infection observed *in vivo*, and to disentangle the response of the different immune and non-immune cell types as well as the molecular pathways involved at specific time-points of the infection. By using diseased bronchial epithelial cells, lung-on-chips can also be designed to model infection in the presence of an underlying chronic disease. For example, Plebani et al. have modeled *Pseudomonas aeruginosa* infection of a cystic fibrosis airway-on-chip (67). They showed that diseased chips contained a higher number of ciliated cells compared to the normal epithelium, and had a higher ciliary beat frequency, which corresponds to prior *in vivo* observations. As expected, the CF chip cells produced more mucus, and also upregulated pro-inflammatory cytokines such as IL-8, while downregulating IP-10, GM-CSF and MIP-1α, indicating an increased inflammatory environment prior to infection. Polymorphonuclear cells (PMNs) introduced in the vascular channel showed an increased adhesion to the endothelial cells and transmigration towards the epithelial channel indicating a heightened infiltration potential, likely in response to signals secreted by the CF epithelium. At 24 hrs. post-infection with *P. aeruginosa*, the bacteria proliferated better in the thick mucus layer of the CF chip, compared to the healthy control and induced the expression of IL-6, TNF-α and GM-CSF. PMNs also showed an increased adhesion to endothelial cells post-infection, which was especially pronounced in the CF chip, however enhanced migration was not observed possibly because they were isolated from healthy donors. An airway-on-chip was also recently used by Laurence Rahme’s group in Aggarwal et al. (68) to investigate the *Pseudomonas aeruginosa* quorum sensing molecule 2’-aminoacetophenone (2-AA) and its transcriptomic effects on the bronchial epithelium and pulmonary endothelium. This molecule had previously been reported by the same group in Bandyopadhaya et al. (69) to direct epigenetic reprogramming of human and mouse macrophages by increasing HDAC1 expression and activity, leading to the global loss of H3K18ac affecting the expression pro-inflammatory cytokines (discussed in more detail below in the section on innate inflammatory memory). In addition, in Chakraborty et al. (70) they reported that 2-AA downregulated lipid biosynthesis and autophagy in macrophages through HDAC1-mediated deacetylation of H3K18ac at the promoters of the *Beclin1* and *Scd1* genes. The airway-on-chip was therefore used as a model to uncover transcriptome-wide effects of 2-AA treatment on lung cells. Differentially expressed genes were observed in both the pulmonary endothelial (4499 DEGs) and bronchial epithelial cells (623 DEGs). Multiple signaling pathways in both cell types were shown to be responsive to 2-AA treatment, either up- or down-regulated, including interleukin, MAPK, TNF, TGF-β, TLR, GPCR, HIF-1, PI3K-Akt, IFN, and receptor tyrosine kinase signaling. Some of the effects were common and some cell type-specific. Interestingly, 2-AA triggered mitochondrial dysfunction in the endothelial cells and cholesterol accumulation in the bronchial epithelial cells, indicating effects on cellular bioenergetics and metabolism, similar to what had been observed previously (71) in macrophages. In addition, this bacterial molecule affected the expression of genes associated with CF and idiopathic pulmonary fibrosis (IPF), such as *CFTR*, *MUC5B*, *MUC4*/*16*, *TGF-β*, *KRT15*/*17*, *CXCL2*/*10*, suggesting that *P. aeruginosa* infection can promote tissue remodeling associated with lung fibrosis or can increase CF severity.

A different type of 3D tissue-engineered human lung model called *AirGels (airway in gels)* was recently used to investigate *P. aeruginosa* biofilm formation (72). What differentiates AirGels from OoCs is that the primary bronchial epithelial cells are grown in the cylindrical cavity of tubular collagen/Matrigel scaffolds at the air-liquid interface and are not under the controlled flow conditions of the airway-on-chip. However, the AirGels contain basal, ciliated and goblet cells and recapitulate the mucus production and mucociliary dynamics observed in the human bronchi. Using AirGels, Rossy et al. (72) elegantly showed that in the very early stages of infection single bacteria attach to the mucus and start to explore the mucosal surface through twitching motions. By 6 hrs post-infection, the bacteria already colonize the mucosal surface and form small interconnected bacterial clusters co-localized with the mucus. Interestingly, the authors observed that the bacteria actively apply force on the mucus using their Type IV pili to contract and remodel it, which encourages bacterial cluster aggregation and eventual biofilm formation (72).

### Advanced models using *in vitro* differentiated cells

Lung organoid-based infectious disease modelling has been used more widely in recent years, especially since refinements in cell isolation procedures, media composition and differentiation conditions have extended the period that organoids can be maintained and expanded *in vitro* (73). Lung organoids are 3D multi-cellular self-assemblies, which contain the major differentiated cell types of the tissue and can recapitulate important aspects of its structural organization and function. They can be derived from primary commercial NHBE cells, from donor-derived bronchus tissue (healthy or diseased), or as more recently reported, from nasal-brushing derived epithelial cells (74, 75). They do not require expensive specialized equipment, making them an accessible yet functional alternative to the more complex OoC described previously. Most of the lung organoid literature is dominated by reports using surface airway epithelial organoids containing the basal, ciliated and goblet cells of the pseudostratified lung epithelial lining, which functions as the lung’s physical and biological barriers against the environment. Recently, submucosal gland organoids (76) have been established containing mucous, serous, basal and myoepithelial cells, which model the mucus-secreting glands of the trachea and large bronchi, and have been shown to contain reserve cells for tissue regeneration (77, 78) following lung injury. Both of these organoid models are indispensable when studying human lower airway infection and inflammation. For example, Pleguezuelos-Manzano et al. (79) used nasal epithelial cell-derived organoids, further cultured in 2D at the air-liquid-interface (ALI) and infected with the wild-type PAO1 strain and quorum-sensing mutants of *Pseudomonas aeruginosa* to investigate gene expression changes occurring in both the host and bacterial cells. They leveraged dual RNA sequencing (80, 81), which allows for the separation of host from pathogen transcripts *in silico*, and performed transcriptomic analysis of the bacteria-organoid co-culture at 14 hrs post-infection. Both the wild-type and QS mutants induced a similar immune response from the epithelial cells, which was enriched with genes involved in cell-cell signaling, cell migration, response to biotic stimulus, response to LPS and immune response (79). However, no clear quorum-sensing effect on the epithelium could be identified, which underscores the point that using bacterial mutants for specific pathways or virulence factors might hinder the identification of significant transcriptome-wide effects, in contrast to using chemically defined treatments with effector molecules. Because the authors sequenced not only RNA from co-culture, but also bacteria in monoculture they were able to observe clear gene expression differences, which as expected, reflected differences in bacterial metabolism between the two culture conditions (79). Following infection of epithelial organoids, PAO1 bacterial cells upregulated genes involved in peptide, glycolipid and amide biosynthetic pathways, as well as genes involved in denitrification. Interestingly, they also upregulated genes involved in antibiotic resistance, such as efflux pump genes (*mexX*, *mexY*, *muxA*), membrane proteins or porins (*oprB*, *oprG*, *oprL*, *oprN*) and antibiotic degrading enzymes (*aph*, PA5514), even though the experiments were performed in antibiotic-free culture conditions (79). This indicates that even at the early stages of infection *P. aeruginosa* upregulates mechanisms for protection against xenobiotics. In addition, the co-culture conditions induced genes from the bacterial Type 3 (for example, *psc* genes) and Type 6 secretion systems, which are used to inject toxic effector molecules into prokaryotic and eukaryotic cells in furtherance of host antagonism and competition between bacteria (79).

As a final example, bronchial epithelial progenitor cells can be differentiated in static conditions at the air-liquid-interface on Transwell inserts, using commercially available differentiation media in a protocol that takes ~4 weeks to complete. This results in pseudostratified airway epithelium ALI cultures, which are relatively easy to handle by less experienced researchers and contain the ciliated, goblet and basal cells of the bronchial epithelium. Work including fluorescence and scanning electronic microscopy imaging, recently reported by Swart et al. (82), has demonstrated the infection kinetics of the PAO1 strain of *Pseudomonas aeruginosa* and the strategy the bacteria use to breach the outer tissue barrier. The authors showed that during the first hours after infection, the bacteria replicate in the mucus layer without much contact with the epithelium. Through a process of asymmetric cell division controlled by the second messenger cyclic diGMP, one motile and one surface-adherent daughter bacterial cell were generated. This process promotes epithelial surface dissemination and colonization such that by 12 hrs post-infection the epithelial surface was covered with bacteria and by 18 hrs the epithelial layer was completely breached (82). Between 6 and 12 hrs post-infection, the authors observed that bacteria were being internalized at a high frequency, overwhelmingly by the non-phagocytic goblet cells. This process was dependent on the T3SS, as well as the H2- and H3-T6SS since mutant bacteria lacking *pscC*, *tssL2* or *tssM3* invaded the goblet cells less efficiently. Interestingly, when mutant T3SS bacteria invaded goblet cells, they localized to vacuoles and continued to divide without significant cytotoxicity, while wild-type bacteria were able to quickly depart the vacuole, localize to the cytoplasm and kill the cell, resulting in their release into the environment (82). This observation illuminates a mechanism which T3SS mutants can use to evade antibiotic therapy and potentially immune surveillance by residing and proliferating inside goblet cells. Through the process of goblet cell invasion and rupture, wild-type bacteria actively breach the surface epithelial barrier of the lung and reach its basolateral side, promoting infection dissemination deeper into the tissue.

**Figure 4:**
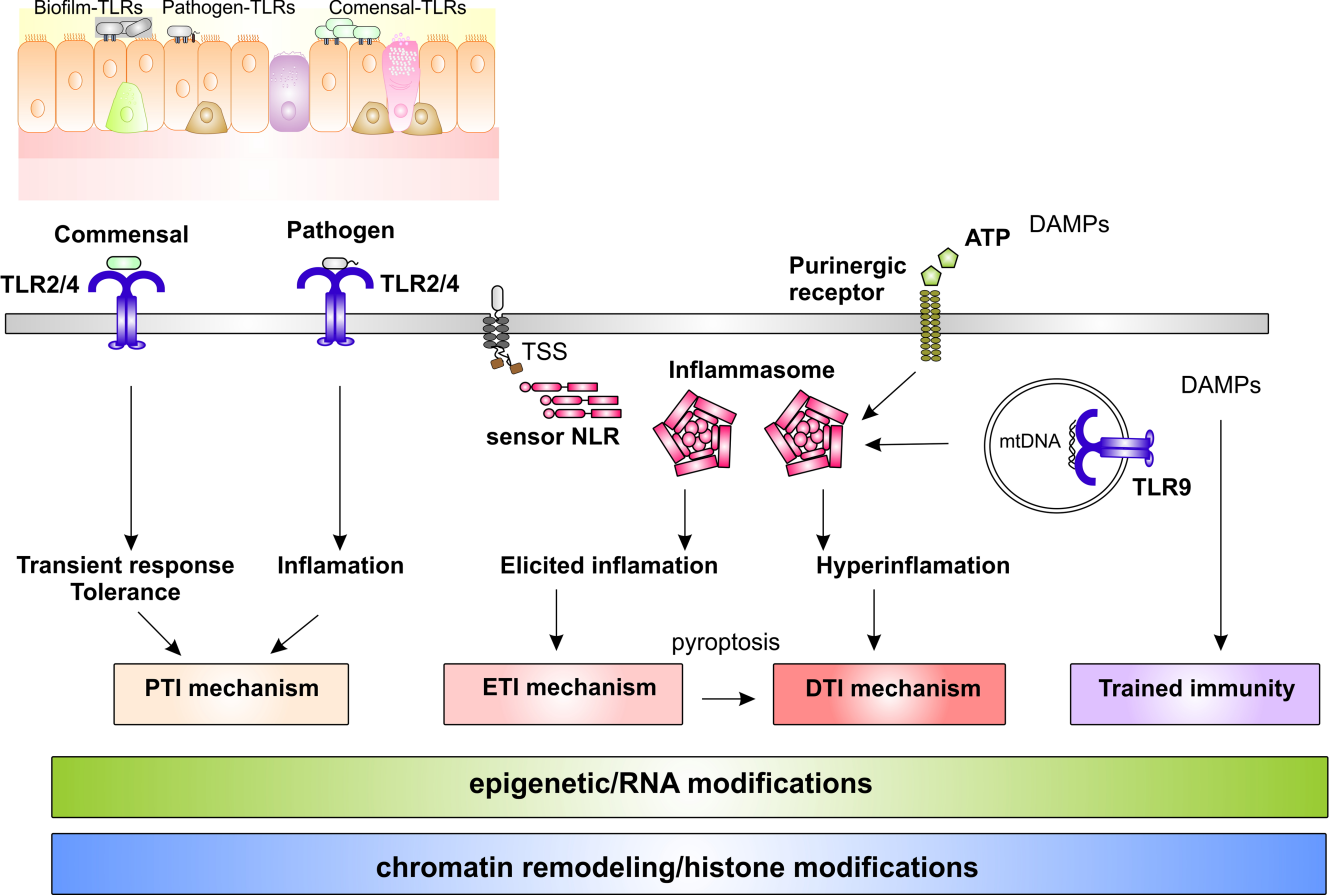
Innate immunity recognition scenarios regulated through epigenetic and/or epitranscriptomic mechanisms. Commensal bacteria and pathogens are recognized by shared innate immune receptors. While commensals induce a transient response with tolerance, pathogens provoke an inflammatory response (pattern recognition receptor-triggered immunity, PTI). Pathogen-derived molecules (toxins, proteins, and nucleic acids) can be sensed by intracellular receptors, followed by formation of the inflammasome, which elicits inflammation via effector-triggered immunity (ETI). ETI can induce metabolic changes and cellular pyroptosis, enhancing the generation of DAMPs (such as mtDNA and secreted ATP), and triggering hyper-inflammation. If the pathogens fail to activate ETI, host cells can be trained by DAMPs to increase fitness upon homologous or heterologous secondary challenge. ATP, adenosine tri-phosphate; DAMPs, damage-associated molecular patterns; mtDNA, mitochondrial DNA; NLR, NOD-like receptors; TLR, Toll-like receptors; ETI, effector-triggered immunity; DTI, damage-triggered immunity; PTI, pattern recognition receptor-triggered immunity.

The *in vitro* models discussed above can aid our understanding of epitranscriptomic dynamics during human lung infection and help us reveal molecular mechanisms that control host cell adaptations to the microbial stimuli. One such adaptation, innate immune memory, is part of a plethora of mechanisms (summarized in **Figure 4**), that work together to provide immediate responses as well as persistent functional changes, which can be beneficial but also detrimental to the organism. Through its foundation in epigenetic reprogramming, it is likely that it intersects with epitranscriptomic regulation. We discuss innate immune memory in the section below.

## Innate inflammatory memory

A body of work in the last decade or so has demonstrated that primary microbial or non-microbial stimuli [for instance, BCG vaccination or sepsis; or exposure to β-glucan, lipopolysaccharide, DAMP molecules such as uric acid or oxidized LDL, and inflammatory cytokines] can cause innate immune cells to change their functional response towards secondary stimulation (for a comprehensive review, please see Bekkering et al. (83)). This phenotypic change can be observed months or even years later, long after the primary signal is gone and cell turnover has occurred. A *bona fide* definition of epigenetically regulated cellular reprogramming (**Figure 4**). This functional change, manifested by enhanced or lowered expression of cytokines, chemokines and other mediators of inflammation in response to re-stimulation is referred to as trained immunity or tolerance, respectively.

The term *trained immunity* was first proposed as a non-specific innate host defense mechanism against reinfection (84). Non-specific innate immune memory has been observed in a variety of prototypical innate immune cells, such as NK (85, 86), macrophages (87, 88), and monocytes (89, 90), and more recently in non-immune cells (e.g. skin (91) and lung (92) epithelial progenitors, coronary smooth muscle cells (93) and fibroblasts (94)). Canonical trained immunity phenotypes are established and maintained independently from adaptive immune cells. However, at least one study has shown the role that CD8+ cells can play in the non-canonical initiation of this process in tissue-resident alveolar macrophages, achieved through the release of effector T-cell derived IFN-γ following a viral infection (88). From a physiological point of view, *training* confers non-specific protection against reinfection with the same or a different pathogen by mounting an enhanced anti-microbial response for more efficient microbial clearance. In contrast, *tolerance* (for example, the immunoparalysis observed post-sepsis) can predispose to future infections by making hematopoietic stem and progenitor cells resistant to mobilizing growth factors, such as granulocyte colony-stimulating factor (G-CSF), and thus unable to activate emergency granulopoiesis (95). At the molecular level, innate immune memory involves metabolic and epigenetic reprogramming with persistent changes in specific histone modifications and chromatin accessibility at particular genomic regions serving as tale-tell signs of training or tolerization (96). This reprogramming results in an increased or impeded transcriptional response to a future immunogenic stimulus. A seminal study by Ostuni et al. provided early insights into the mechanisms by which this type of cellular reprogramming can occur (97). It found that terminally differentiated cells possess transcriptional plasticity in response to transient external stimulation and subsequent re-stimulation, due to the unmasking of latent, previously inactive, enhancer regions. When the cells are in their basal functional state, these cis-regulatory elements have no histone modification marks and are not bound by transcription factors (TFs). However, following stimulation they gradually acquire H3K4me1, H3K27ac and become accessible for binding by both lineage-determining and stimulus-induced TFs (97). After the primary stimulus is gone, these enhancers retain residual H3K4me1 and upon secondary stimulation drive faster induction and higher expression of the genes they control, which are generally located close by (97). This and other studies have shown that the strength, type and duration of the primary stimulus, the receptors it engages and the signaling pathways it activates all affect which downstream genes will be responsive (89, 96, 97). In some cases, LPS being an example, the concentration of the same stimulus determines whether it leads to training, or tolerization (89).

Not only structural bacterial components, such as flagellin, muropeptides or LPS, but also secreted virulence factors such as quorum sensing molecules can have immunomodulatory functions and be involved in establishing innate inflammatory memory. Of these, 2’-aminoacetophenone (2-AA) is an especially interesting *Pseudomonas aeruginosa*-derived secreted small molecule, whose expression is controlled by one of the QS master regulators MvfR (PqsR). 2-AA has been demonstrated to epigenetically rewire human and murine immune cells, resulting in immunosuppression (69–71, 98). Bandyopadhaya et al. have shown that single stimulation of human monocytes and mouse macrophages with 2-AA induced the expression of TNF-α, IL-1β and MCP-1 at the gene and protein levels (69). However, repeated treatment with 2-AA blunted that response. Mechanistically, single 2-AA stimulation leads to the accumulation of H3K18ac, but not H3K9ac or H3K9me3 in two promoter regions of *Tnf* and also globally, however, repeat treatment results in the loss of H3K18ac. By using histone deacetylase (HDAC) inhibitors with different specificities, the authors showed that repeat treatment with 2-AA drastically increased HDAC1 protein abundance, enzymatic activity and binding to the *Tnf* promoter (69). Utilizing an *in vivo* burn model of *Pseudomonas aeruginosa* infection, repeat treatment with 2-AA was shown to increase the survival rate of the mice, irrespective of higher bacterial loads. This beneficial effect was lost if the mice were also treated with trichostatin A (TSA), an HDAC inhibitor with broad specificity, indicating that it required histone deacetylase activity (69). This study showed that both *in vitro* and *in vivo*, 2-AA can serve as an immunomodulatory effector molecule by reducing the strength of the innate immune response to infection, which promotes bacterial survival and persistence, but is also beneficial for the host by allowing it to tolerate a chronic bacterial infection. A follow-up study showed that in macrophage cells, in which tolerance was induced with 2-AA, the levels of NF-κBp65 acetylation at position K310 were decreased resulting in its reduced transcriptional activity (98). The same was true for the auto-acetylation site of CBP/p300 at K1499, which resulted in its reduced lysine acetyl-transferase (KAT) activity. The loss of these critical PTMs, concurrent with the increased HDAC1 abundance in 2-AA tolerized cells led to a preferential interaction between HDAC1 with NF-κBp50 and resulted in transcriptional silencing of inflammatory genes through the loss of CBP/p300-mediated H3K18ac at their promoters (98). It should be noted that CBP/p300 is a KAT with an exceptionally broad acetylome repertoire, which includes histone proteins, but also non-histone targets (99). Just to name a few, these include a diverse set of regulatory proteins: i) regulators of transcription and enhancer activity, such as PTEF-b, MLL3/4 and MEDIATOR complexes; ii) regulators of chromatin organization, such as the Cohesin complex; and iii) transcription factors involved in multiple signaling pathways, including but not limited to Notch, Wnt, TGF-β, Nuclear hormone receptor signaling and circadian rhythm regulation (99). It is therefore not surprising that 2-AA exhibited such broad transcriptomic effects on lung cells in Aggarwal et al. (68) (discussed previously in the subsection on organ-on-chip models of infection). Our expectation is that future research on 2-AA will continue to reveal its extensive effects on multiple layers of gene expression regulation that affect many cellular functions, depending on the cell context.

As mentioned earlier, elevated serum lactate levels are observed in sepsis, with higher lactate associated with increased disease severity, septic shock and higher mortality rates. In addition to its acetyl-transferase activity, CBP/p300 also acts as a lactyltransferase (45) specifically at H3K18, which has recently been shown to be a histone mark for cell type-specific active enhancers (100). Given the relevance of H3K18la and H3K18la/ac levels to sepsis discussed earlier, it is especially intriguing that Ziogas et al. have recently shown that human trained innate immune cells, such as monocytes and macrophages, use H3K18la enriched at distal regulatory elements to mark genes that are poised to respond to secondary stimulation (101). Their remarkable study links the metabolic reprogramming characteristic of innate immune cell training to the global epigenetic reprogramming that underlies the robustness of the transcriptional response to re-stimulation (101). Moreover, the study expands the list of training-associated epigenetic marks, adding H3K18la to H3K27ac, H3K4me1 and chromatin accessibility, and shows the modification’s stability *in vivo* by demonstrating its persistence in human monocytes up to 90 days post-stimulation of volunteers with BCG vaccination (101). Among their many insights, the authors also establish p300 as a primary mediator of training through its histone lactylation activity, and also suggest inter-individual differences in the strength of innate immune training linked to polymorphisms in genes for *EP300* and Lactate dehydrogenase A (*LDHA*) (101). As mentioned previously, METTL3 gene expression can be induced by H3K18la of its promoter, suggesting a possible intersection between H3K18la reprogramming during innate immune memory and METTL3-mediated m6A modification of transcripts for regulatory proteins.

## Discussion

Pathogenic Gram-negative bacteria continue to be of high-priority due to their ubiquity as well as their adaptive nature and opportunistic behavior. In line with the effecter-triggered immunity concept, these pathogens can exploit various host arsenals, including multiple sensors or cell guardrails as well as RNA modifying mechanisms, in order to escape from immune defense or unleash inflammation. Hypothetically, ETI processes may also be important for the prevention of antibiotic resistance. Indeed, bacteria frequently acquire antibiotic resistance through horizontal gene transfer, but they also possess intrinsic resistance mechanisms. Predicting resistance is essential for effectively combating bacterial infections. Recently, Khaledi et al. developed a machine learning approach for predicting *P. aeruginosa* antimicrobial resistance using genomic and quantitative gene expression information from 414 clinical isolates (102). Their study did not identify gene expression profiles or polymorphisms within genes for RNA modification enzymes as markers for resistance against four antibiotics (tobramycin, ciprofloxacin, meropenem, and ceftazidime) (102). However, it will be interesting to see whether or not the addition of RNA modification information to the model can enhance its predictive value even further, especially for antibiotics whose mechanism of action involves binding to rRNA. Going forward, powerful sequencing technologies that can simultaneously provide gene expression and RNA modification information, as well as their adaptation to clinical settings will be a valuable tool in this regard (103).

De Crecy-Lagard et al. have suggested that certain bacterial processes are controlled through evolutionarily conserved regulation of translation speeds, which makes them particularly sensitive to tRNA modification dysregulation (104). For example, bacterial motility (either reduced, or increased), amino acid metabolism (particularly affecting histidine) and iron homeostasis were consistently altered in mutants for a variety of tRNA-modifying enzymes from different bacterial species, and the authors demonstrated that these were cellular processes regulated by translation speed and/or ribosome pausing (104). Interestingly, transcriptomic data for some *E. coli* mutants affecting core tRNA modifications cluster together, as did transcriptomic data for mutants affecting anticodon tRNA modifications (104). This indicates that functionally similar tRNA modification defects (i.e. influencing tRNA stability vs. translation fidelity) trigger similar cellular outcomes that elicit similar global gene expression changes in response to the induced stress. As the case of MiaB in *P. aeruginosa* shows, tRNA modifying enzymes can affect bacterial physiology indirectly, but can also integrate signals from an extended protein network with translational outcomes a direct result of tRNA hypomodification.

Much of what we know about *Pseudomonas aeruginosa* biology and the host’s response to acute infection comes from mouse models infected with laboratory strains PAO1 and PA14, and from *in vitro* cultivated bacteria. However, these are inadequate to investigate how the pathogen and human cells interact during chronic infection, and especially when we are interested in RNA modifications, which change dynamically in response to nutrient availability and environmental stress. Physiologically relevant *in vitro* human airway models give us experimental access to the earliest stages post-infection in strictly controlled environments and at high spatiotemporal resolution, which is unfeasible with animal models. They also allow for the application of chemically defined treatments with bacterially-derived effector molecules, which increases signal-to-noise ratios in complex (epi)transcriptomic and epigenomic data, where effect sizes might be small and masked by massive responses to LPS, flagellin and other traditionally investigated bacterial factors. Bacterially-derived effector molecules, structural components and metabolites can trigger profound and long-term epigenetic and metabolic reprogramming in immune and non-immune cells, in order to tolerize the host towards persistent infection or to train it for increased fitness in the face of secondary challenge. Whether or not RNA modification plays a causal or supportive role in these adaptation mechanisms is yet to be determined.

## Conclusion

The epitranscriptomics field is maturing. Yet, a small number of modifications have been mapped at single-nucleotide resolution across diverse RNA species. Multiple studies discussed here report results from RNA immunoprecipitation, which depends on antibodies that are not readily available. RIP-based approaches are unable to uncover precise modification locations - a significant drawback in the context of position-dependent functions. A further limitation is the siloed nature of the research. RNA modifications are frequently investigated one at a time, instead of comprehensively. Wider adoption of methods such as direct RNA nanopore sequencing, which has the potential to detect multiple modifications on single transcripts, and its adaptation to low-inputs will be beneficial. In this way the *RNA modification code*, similar to the histone code in epigenetics, can be revealed. Integrating epitranscriptomic with other Omic data also remains insufficient, and we might be missing how and where the different layers of gene expression regulation reinforce each other. Studies of RNA modifications in infectious disease should take care to streamline their design in order to facilitate data comparison, especially when precious patient samples are involved. The field of epitranscriptomics can better leverage validated advanced human near-physiological *in vitro* models, which can overcome the complexity and noise of animal model systems. On the microbial side, the overwhelming use of adapted bacterial laboratory strains is hindering the discovery of biologically relevant mechanisms. Moving beyond simple associations, there should be a concerted effort to definitively prove whether or not bacterial phenotypes, such as changes in pathogenicity or antimicrobial resistance, are due to RNA hypomodification. In summary, the RNA modifications field is growing rapidly and holds great promise for the development of new types of therapeutic agents with wide application in inflammatory and infectious disease, but significant hurdles remain.
